# Sensitivity of the RNA Structure to Ion Conditions as Probed by Molecular Dynamics Simulations of Common Canonical RNA Duplexes

**DOI:** 10.1021/acs.jcim.2c01438

**Published:** 2023-03-29

**Authors:** Petra Kührová, Vojtěch Mlýnský, Michal Otyepka, Jiří Šponer, Pavel Banáš

**Affiliations:** † Regional Centre of Advanced Technologies and Materials, Czech Advanced Technology and Research Institute (CATRIN), Palacký University Olomouc, Šlechtitelů 27, 779 00 Olomouc, Czech Republic; ‡ Institute of Biophysics of the Czech Academy of Sciences, Královopolská 135, 612 00 Brno, Czech Republic; § IT4Innovations, VSB − Technical University of Ostrava, 17. listopadu 2172/15, 708 00 Ostrava, Poruba, Czech Republic

## Abstract

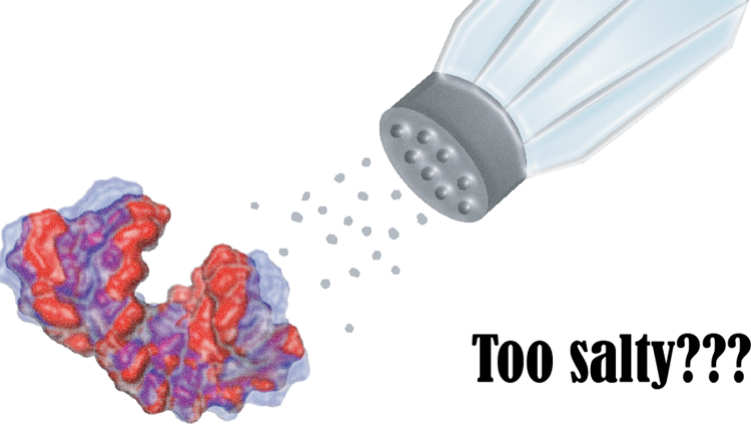

RNA molecules play a key role in countless biochemical processes. RNA interactions, which are of highly diverse nature, are determined by the fact that RNA is a highly negatively charged polyelectrolyte, which leads to intimate interactions with an ion atmosphere. Although RNA molecules are formally single-stranded, canonical (Watson–Crick) duplexes are key components of folded RNAs. A double-stranded (ds) RNA is also important for the design of RNA-based nanostructures and assemblies. Despite the fact that the description of canonical dsRNA is considered the least problematic part of RNA modeling, the imperfect shape and flexibility of dsRNA can lead to imbalances in the simulations of larger RNAs and RNA-containing assemblies. We present a comprehensive set of molecular dynamics (MD) simulations of four canonical A-RNA duplexes. Our focus was directed toward the characterization of the influence of varying ion concentrations and of the size of the solvation box. We compared several water models and four RNA force fields. The simulations showed that the A-RNA shape was most sensitive to the RNA force field, with some force fields leading to a reduced inclination of the A-RNA duplexes. The ions and water models played a minor role. The effect of the box size was negligible, and even boxes with a small fraction of the bulk solvent outside the RNA hydration sphere were sufficient for the simulation of the dsRNA.

## Introduction

Ions are an integral part of the biomolecule environment and play an important structural and functional role that has yet to be fully understood.^1−6^ An important function of ions is to compensate for the biomolecule charge, particularly with nucleic acids that bear a negatively charged backbone. Charge neutralization plays a crucial role in the process of folding nucleic acids by allowing tertiary contacts of negatively charged backbones in space. The negative charge of the polyanionic backbone of nucleic acids is shielded by a sufficient number of metal ions in the condensation layer around the DNA and RNA, and its function is to neutralize the backbone’s negative charge. Counterions can interact either directly with the RNA structure (referred to as chelated ions)^7,8^ or through a shell of coordinated water molecules (referred to as diffuse ions or “ion atmosphere”).^9^ The mobility of chelated ions is low because they are usually tightly bound to RNA. Tightly bound ions, with two or more first shell contacts to RNA, are the least abundant but, in some cases, make an important contribution to specific local or even global conformations. The tightly bound ions are precisely localized due to the discrete interactions with the RNA and cannot be easily replaced by other ions from the solvent.^10,11^

In contrast, diffuse ions are an important factor in the folding, stabilization, and interactions of the RNA tertiary structures due to their vastly larger populations. They act as a charge-neutralizing layer around the RNA.^8^ These ions tend to remain fully hydrated and do not occupy any particular structurally well-defined binding sites, which makes their structural characterization difficult by most biophysical techniques. Their high mobility enables measurement of the distributions but not the positions of individual ions. An experimental approach that has been successfully used to study the diffuse ions is ion counting (IC). This method quantifies the number of thermodynamically accumulated cations and thermodynamically excluded anions around a negatively charged nucleic acid. Experimental methods based on IC include buffer equilibration atomic emission spectroscopy,^12−17^ anomalous small-angle X-ray scattering,^18−21^ and titration with fluorescent dyes.^22^ Ion condensation around nucleic acids can be also studied following theoretical computational approaches. A traditional method for describing ionic solutions is the Poisson–Boltzmann theory, which has been successfully used to predict charge distribution and interactions between the charged biomolecules.^23,24^ This method is a starting point for many theoretical approaches as it is based on a simple and analytically tractable model that can be easily extended and modified. Although the theory has limitations when the ion correlation becomes significant,^25^ it provides meaningful results that are in good agreement with many experiments.^26^ Another theoretical tool for treating the distribution of counterions around highly charged polyelectrolytes is Manning’s counterion condensation theory.^27−31^ This theory suggests that the fixed charges on the rod are neutralized by 0.7–0.8 monovalent cations being condensed onto them, while the non-condensed counterions are distributed according to the Poisson–Boltzmann theory. This approach is useful for rod-like molecules such as DNA but not applicable to non-rod-like biomolecules such as folded RNA. Ion condensation around folded polyelectrolytes can be predicted by non-linear Poisson–Boltzmann equations.^8,32^ Unfortunately, this method does not provide information about the exact distribution of ions around nucleic acids. Atomic details of the ionic interactions of nucleic acids can be obtained adopting integral equation-based molecular solvation methods such as 3D RISM^33−35^ or by molecular dynamics calculations.^20,36−54^

The ionic conditions in cells should be taken into account when modeling *in vivo* conditions by molecular dynamics (MD) simulations. Although a large variety of ions interact directly with RNA in various biophysical experiments, the most biologically relevant ions are K^+^, Na^+^, Mg^2+^, and Cl^–^. This reduces the set of biologically relevant ions for MDs of RNA. Due to the challenges arising from the force field and sampling when describing divalent ions,^55^ MD simulations of nucleic acids are mostly performed solely in the presence of monovalent ions. K^+^ is the major cation found in the cell (*c*_K+_ = 0.15 M), while Na^+^ dominates in extracellular fluids. However, a limited number of studies compared the effects of Na^+^ versus K^+^ cations on the RNA structure. Some of them reported that simulations with net-neutralizing Na^+^ ions and under 0.2 M excess salt conditions seem to be equivalent in all aspects.^56−58^ Other simulations showed that 0.4 M and more excess salt caused a modest sequence-dependent compaction of canonical A-RNA double helices.^59,60^ On the other hand, several studies that tested different conditions such as Na^+^ vs K^+^ and net neutral vs excess-salt simulations found that the simulations are insensitive to the type and concentration of the monovalent ions.^56,61−64^ More likely, the simulation results showed that the systems are more sensitive to the explicit water model.^61,63−65^ Nevertheless, it is possible that the (in)sensitivity to the type of ion is to some extent dependent on the simulated systems. In MD simulations of nucleic acids, anions are generally considered to have a minimal effect on the results, but they are widely used to compensate for the positive charge in the excess salt condition.

**Figure 1:**
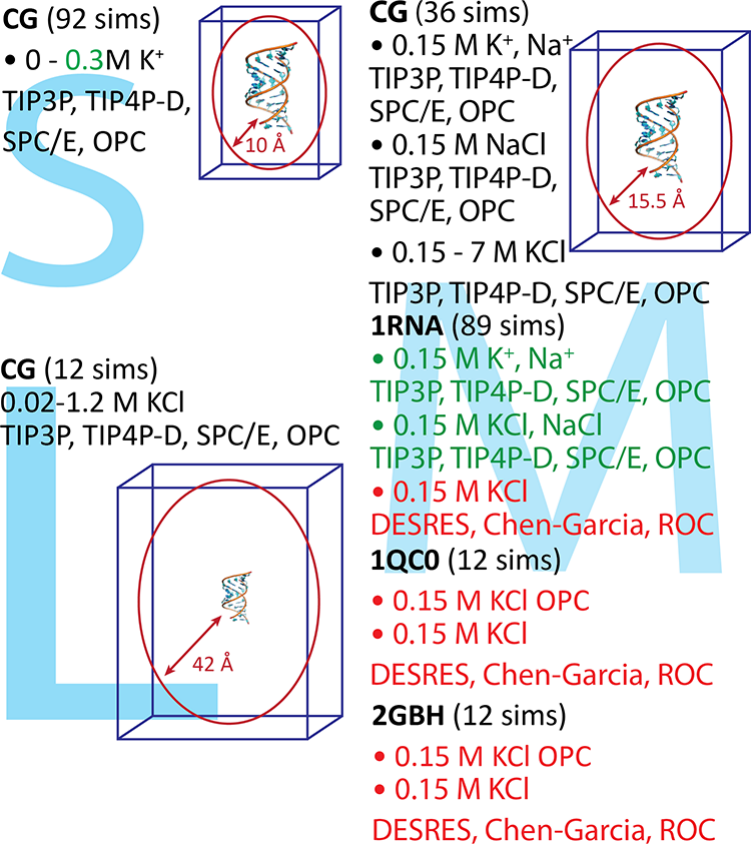
Scheme of all simulations performed in this study together with the information about the simulated system (bold), total number of simulations for each system (“sims”, in parantheses), type and concentration of used ions, used water model and size of solvent box (S, M, and L in the background). The red and green colors indicate simulations run three and five times, respectively. All simulations were run with the OL3 force field, except where another force field is indicated.

In this study, we used all-atom explicit solvent MD simulations and investigated the effect of monovalent counterions and size of the solvent box on the shape of the canonical A-RNA duplexes. We focused on several basic objectives: (i) the effect of the number of ions on the A-form RNA, (ii) the description of the distribution of the ions around the RNA, (iii) the effect of the size of the used simulation box on the RNA structure, (iv) the effect of the used ions and the ion concentration on the RNA molecule, and finally (v) the effect of the used solute (RNA) force field (Figure 1 and Table S1 in the Supporting Information). The RNA A-form helical shape is dominantly affected by the applied RNA force field. Some of the tested force fields appear to underestimate the inclination of the dsRNA, although the experiments do not provide an exact quantitative benchmark data in this respect. The inclination values reported by crystallographic data might be affected by crystal packing and crystalline conditions, while the solution experiments provide only indirect evidence about the inclination, with the refinement procedure possibly biasing the particular reference value. Nevertheless, the ability of a force field to provide a sufficient inclination value of the dsRNA seems to be a specific issue that needs to be considered when parametrizing force fields since a significant reduction in the inclination value may lead to undesirable results of certain force-field modifications. The A-RNA shape is only moderately modulated by the ionic parameters, concentrations, and water model. Among the various tested ionic parameters, the Joung–Cheatham parameters associated with the SPC/E water model have a stronger tendency than the other tested ions to drive the RNA duplexes into more compact helical structures. Although we have studied only a limited set of sequences, we confirm that the shape of the canonical A-RNA was affected by the RNA sequence. What remains unexplored and should be addressed by further research is how well the simulations can determine the sequence effect on A-RNA properties that can be characterized by experimental tools.

## Methods

### Starting Structures and the Basic Simulations Setup

Most of the simulations were carried out using the AMBER 16 program package^66^ with the standard OL3^67−70^ RNA force field. The OL3 force field was augmented by the vdW modification of phosphate oxygens developed by Steinbrecher et al.;^71^ all the affected dihedrals were adjusted as described elsewhere.^72^ The AMBER library file of this force field version can be found in the Supporting Information of Ref (73). In addition, we applied the external generalized interaction-specific gHBfix potential, which has been shown to improve the performance of the small RNA oligonucleotides and to avoid any side effects on both small and more complex RNA systems.^74,75^ The setup for the gHBfix was as follows: (i) all NH–N hydrogen bond (H-bond) interactions were supported by 1.0 kcal/mol and (ii) all OH–nbO H-bond interactions were weakened by −0.5 kcal/mol, for more information see Ref (75). The performance of the force field for A-RNA double helix is assumed to be primarily determined by the core OL3 part of the force field. Previous tests have shown that the effect of the gHBfix on the inclination of dsRNA is rather minimal.^74,75^

The starting structures of the three RNA double helices were taken from 1RNA(76) and 1QC0(77) crystal structures at a resolution of 2.25 and 1.55 Å and from the 2GBH(78) NMR structure (first frame), respectively. The 1RNA structure had mostly alternating UA sequence r[U(UA)_6_A]. From the 1QC0 duplex, 10 base pairs were used with sequence r(GCACCGUUGG). The 2GBH structure contained eight base pairs with sequence r(GGGCUAAU). The fourth system containing alternating CG base pairs was initially built using Nucleic Acid Builder of AmberTools^79^ with sequence r(CG)_5_. We note that the simulations of the canonical dsRNAs initiated from the X-ray or NMR measurements and the built-up structures were equivalent (except for the initial short period).

All RNA systems were immersed into a solvation box of TIP3P,^80^ SPC/E,^81^ OPC,^82^ and TIP4P-D^83^ explicit water molecules with a depth of 15.5 Å on each side of the solute. The parameters of the used ions for each explicit water model were as follows: K^+^ (TIP3P: *r* = 1.705 Å, ε = 0.1936 kcal/mol, SPC/E: *r* = 1.593 Å, ε = 0.4297 kcal/mol, OPC: *r* = 1.590 Å, ε = 0.2795 kcal/mol, TIP4P-D: *r* = 1.76375 Å, ε = 0.087 kcal/mol),^84^ Na^+^ (OPC: *r* = 1.2260 Å, ε = 0.1684 kcal/mol),^84^ and Cl^–^ (TIP3P: *r* = 2.513 Å, ε = 0.0355 kcal/mol, SPC/E: *r* = 2.711 Å, ε = 0.0127 kcal/mol, OPC: *r* = 2.760 Å, ε = 0.0117 kcal/mol, TIP4P-D: *r* = 2.27 Å, ε = 0.15 kcal/mol).^84^ For the description of the simulation set-up, see the Supporting Information. All simulations were run with a 2 fs integration step on the 1 μs time scale, which proved to give sufficient convergence for the A-RNA duplexes.^59^

### Box Size

As a model system for testing the box size and ionic concentrations, a built-up A-RNA helix was chosen, consisting of 10 base pairs with a repetitive CG sequence – r(CG)_5_. The CG-tract was used on the basis of our previous works where we showed that it was the most sensitive one to the choice of the force fields and the ionic strength.^59^ The three different sizes of the simulation box were prepared using variable minimal distance between the solute and the edge of the periodic box. The distance between the outmost nucleic atom and the closest simulation box face varied from 10 Å (here, the simulations denoted as S) to 15.5 Å (denoted as M) to 42 Å (denoted as L). Further, we changed the concentration of the ions in the solvation boxes (for the overview of all the simulations, see Figure 1 and Table S1). The smallest boxes denoted as S were simulated with counterions varying from 0 to 18 (18 K^+^ ions corresponded to net-neutral conditions). For simulations with the uncompensated charge (i.e., simulations with 0–17 K^+^ ions), a uniform charge density was used to neutralize the simulation box. In addition, the simulation using small boxes was run five times under net-neutral conditions in order to check convergence. The box denoted as M was simulated using varying ionic concentrations ranging from 0.15 to 7 M KCl (no salt crystallization occurred even at the highest concentration) and finally the boxes called L were used with three different concentrations: L1 contained 18 K^+^ ions (net-neutral conditions), L2 involved 120 K^+^ and 102 Cl^–^ ions (physiological concentration of ions), and L3 included 933 K^+^ and 915 Cl^–^ ions. The schematic view of all simulations used in this study together with the information about simulated system, ion type, used water model, and size of solvate box is shown in Figure 1. All solvation boxes were simulated using four different explicit water models – TIP3P, SPC/E, TIP4P-D, and OPC. TIP3P and SPC/E were used because of their high popularity and relatively low computational cost. The other two models were chosen because their parameters describe the properties of water more accurately. In addition, we prepared two hybrid simulations of a CG duplex by swapping the ion parameters for the TIP3P water with the ion parameters for the SPC/E water model, i.e., one simulation with ions for the TIP3P water model simulated with the SPC/E water box and vice versa.

### Testing of Na^+^ vs K^+^ of Ions

To test the influence of different types of ions, we performed a series of simulations of the CG duplex and the 1RNA duplex with different explicit water models. The simulations were run under net-neutral (0.15 M) Na^+^ or K^+^ conditions or under 0.15 M NaCl or KCl excess salt.

### Testing of Solute Force Fields

The OL3 force field is currently the most widely used and tested RNA force field. We also performed some additional simulations as follows: (1) A set of simulations of all the abovementioned duplexes using a force field developed by Aytenfisu et al.^85^ (hereafter abbreviated as ROC *ff*) with 0.15 M KCl in combination with TIP3P water model (K^+^: *r* = 1.705 Å, ε = 0.1936 kcal/mol, Cl^–^: *r* = 2.513 Å, ε = 0.0355 kcal/mol),^84^ (2) a set of simulations of 1RNA, 1QC0, and 2GBH using the Chen–Garcia force field^86^ with 0.15 M KCl and the TIP3P water model (K^+^: *r* = 2.658 Å, ε = 0.000328 kcal/mol,^86,87^ Cl^–^: *r* = 1.948 Å, ε = 0.265 kcal/mol^88^), and (3) a set of simulations of 1RNA, 1QC0, and 2GBH using force field published by Shaw and co-workers^89^ (hereafter abbreviated as DESRES *ff*) with 0.15 M KCl and the TIP4P-D water model (K^+^: *r* = 1.76375 Å, ε = 0.087 kcal/mol, Cl^–^: *r* = 2.27 Å, ε = 0.15 kcal/mol).^90,91^ It should be noted that the aim of our work was not to provide a full test of all available force fields. Our objective was to obtain a basic insight into the sensitivity of the dsRNA simulations of the RNA force field, rather than to perform an extended test of RNA force fields. The present data can be easily used as a benchmark for any other force field versions.

### Performed Analyses

The global behavior of each system and the density maps of counterions were analyzed with the *cpptraj* module (AMBER tool).^92^ Prior to the analysis of the density maps, each system was centered, imaged, and RMS fitted, and finally the density of the ions was calculated using cubic grids distributed over the entire box volume. The output file was visualized using the VMD^93^ program (http://www.ks.uiuc.edu/Research/vmd/). The 2D ion concentration maps around the RNA were calculated from the counterion density maps by being projected onto the *x–y* plane through the central part of the CG duplex in the *z* axis (i.e., for the part containing G4-C17, C5-G16 and G6-C15 base pairs). It should be noted that these concentration maps may have been biased by the selection of the part of the duplex over which the *x–y* projection was made. Further, the *cpptraj* module was used for monitoring the distances between the atoms, end-to-end distances, dihedral backbone angles, sugar pucker, and helical parameters. Because of the higher flexibility of the nucleotides at the end of the helices, the helical parameter analysis omitted two terminal base pairs at both ends of the studied structures. The frequency of the base pair opening (base pair fraying) of the terminal residues among different simulation settings was mapped separately. The base pair fraying at the helix termini was monitored using an in-house program for the identification and classification of the type of the base pair family. The program automatically identifies and classifies all types of RNA base pairs formed by various combinations of the three edges. The concentration radial distribution function was calculated from minimal RNA-ion distances from all surface atoms with 0.1 Å binning by an in-house program using non-imaged trajectories. Both in-house programs are part of the Supporting Information. Moreover, we analyzed the nuclear Overhauser effect intensities for the 2GBH duplex. We report the χ_NOE_^2^values as the difference between the calculated and the experimental NOEs, assuming Gaussian experimental errors, i.e.,
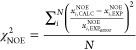
where *N* is the total number of analyzed experimental NOEs^78^, *x*_*i*, EXP_error__^NOE^ is the higher from the reported (*x*_*i*, EXP_max__^NOE^ – *x*_*i*, EXP_^NOE^, *x*_*i*, EXP_^NOE^ – *x*_*i*, EXP_min__^NOE^) values, and *x*_*i*, CALC_^NOE^ are averages over the *M* samples obtained from MD simulations, i.e., . The lower the total χ_NOE_^2^ value, the better agreement between the simulation ensemble and the experimental data (χ_NOE_^2^ values below 1 indicate good agreement with the experiment).

## Results and Discussions

We report a large number of standard MD simulations using four A-RNA duplexes with a total simulation time of over 250 μs. To simplify the presentation, we focused our analyses on the compactness of the A-RNA structure, which is best (and dominantly) described by the inclination of the base pairs with respect to the helix axis.^59,61,64^ In contrast to end-to-end distance, which may also be used as a measure of the A-RNA helix compactness, the inclination is not sensitive to the terminal base pair fraying and therefore provides a more accurate insight into the A-RNA helix compactness (see Table S3). Some other helical parameters and end-to-end distances are documented in the Supporting Information. We note that the inclination is mathematically directly coupled to the base pair roll through a helical twist, so that the inclination and the base pair roll mutually correspond with the helical parameters that are defined in the global and local coordination frames, respectively. A-RNA inclination very correlates with helical rise.^61^

### Single 1 μs Simulation Is Sufficient to Characterize the A-RNA Compactness

**Figure 2:**
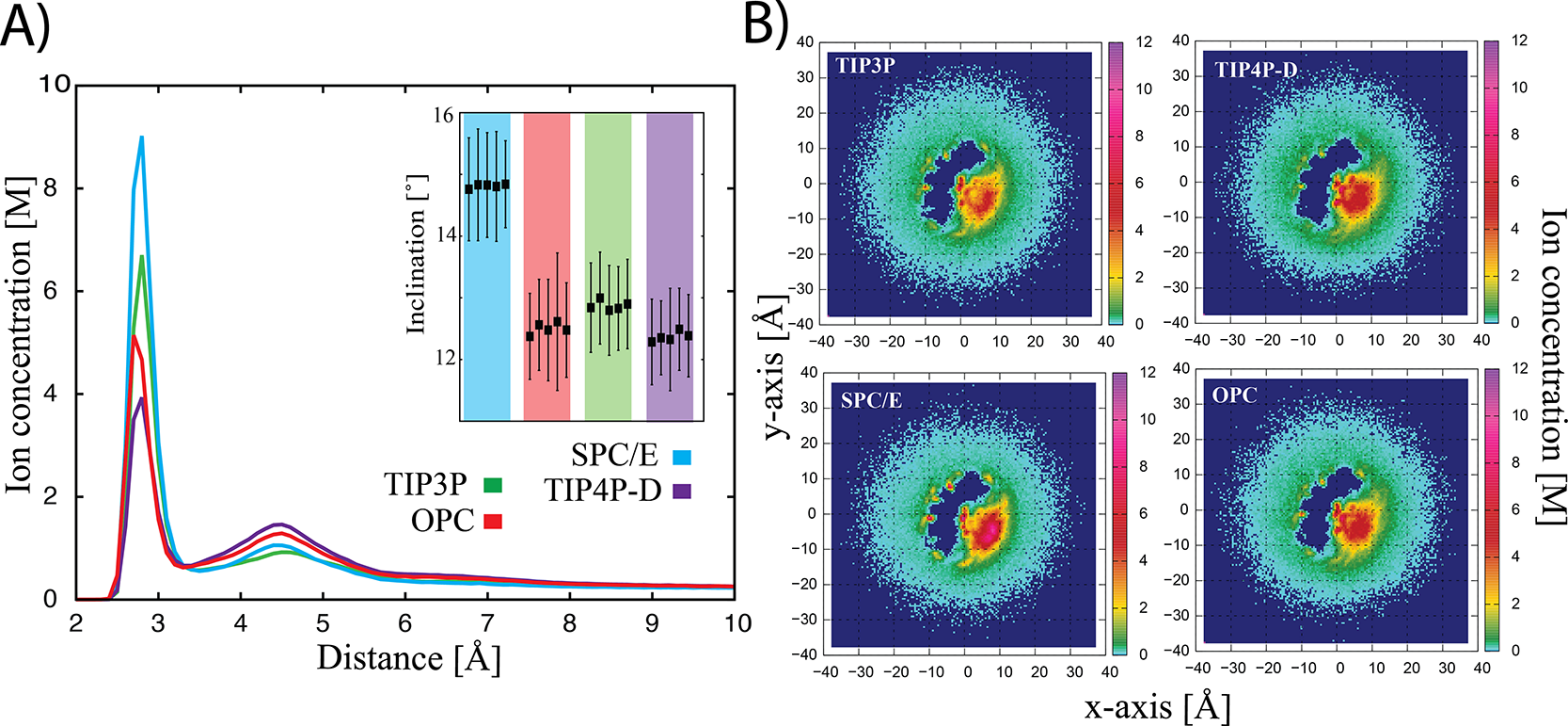
(A) Radial concentration profiles of ions calculated for net-neutral S-box simulations of the CG duplex as a function from the center of the RNA. For clarity, only one simulation from multiple simulations is shown as the results are almost identical for all simulations (see Table 1 for the number of ions in the first two solvation shells). The inset shows the mean values of the inclination (and standard deviations represented by error bars) of multiple CG duplex simulations calculated for the six innermost base pair segments. (B) 2D ion concentration (K^+^) maps of the CG duplex simulations corresponding to the simulations in section A. The lower right corners correspond to the major grooves and the upper left corners correspond to the minor grooves.

First, we performed a series of multiple simulations of the CG-tract using a small box (referred to as S-box, see Figure 1) under net-neutral condition to assess the variation of the RNA structural parameters in the dataset. The number of parallel MD simulations in each subset was chosen according to Day and Daggett,^94^ who suggested that 5–10 simulations are sufficient to assess the variance in the data. The TIP3P, TIP4P-D, and OPC water models showed similar inclination values (varying between 12.3° and 12.7°, see the inset in Figure 2). The SPC/E water model increased the inclination by almost 2°. The variation of the inclination parameter between multiple equivalent simulations was less then ∼0.2° (see inset of Figure 2). It confirms that a single 1 μs simulation provides statistically significant results for A-RNA. This is related to the fact that the A-RNA double helix samples dominantly just one backbone conformation, in contrast to B-DNA with its BI/BII substates.

In order to obtain an insight into how ions were arranged around the A-RNA, we calculated radial concentration profiles of ions and 2D ion concentration maps of the central part of the CG duplex. In all cases, the cation RDFs were characterized by two well-structured peaks indicating the formation of two ionic shells around the A-RNA with counterions accumulated within ∼8 Å of the RNA surface. These two peaks were attributed to specific ion-binding sites and structural features of the A-RNA. The first major peak correlated with the ions bound to the major and minor grooves. The second (minor) peak corresponded to the binding of ions to the backbone oxygens. The comparison of the radial concentration profiles for different water models (see Figure 2A and Table 1) showed that the positions of the first and second peak were similar for all water models and the main difference between the water models consisted in the height of both peaks, which correlated with the number of ions bound to the RNA. The total sum of ions bound in the first and second shells was quite similar for the OPC, TIP4P-D, and TIP3P models (i.e., four to six and 11 or 12 bound K^+^ ions in the first and second shells, respectively, Table 1). The trends seen in the RDF for individual water models were also supported by the 2D ion concentration maps (Figure 2B). The SPC/E model showed the highest density near the RNA in both minor and major grooves. The TIP3P model showed a higher density in the large vicinity of the RNA, corresponding to the first solvation shell, but the concentration decreased sharply with distance. The TIP4P-D and OPC models showed very similar results, with the OPC having a higher density than the TIP4P-D in the closest proximity to the RNA, which corresponded to the first peak of the RDF. The RDF analyses and ion concentration maps suggested that the effect of the used explicit water models on the ionic shells around the RNA followed the same trends as for the values of the inclination. Thus, the SPC/E model again deviated significantly from the other water models, showing a twofold higher population of ions in the first solvation shell than the OPC and TIP4P-D models. On the other hand, the total sum of ions in the first and second shells in the SPC/E simulations was relatively comparable to other water models (i.e., 11 or 12 compared to 13 or 14 tightly bound K^+^ ions).

**Table 1:** Number of Ions in the First and Second Solvation Shell for All Net-Neutral S-Box Simulations of the CG Duplex^a^

		first shell		second shell	
water	#sim	(Å)	N	(Å)	N
TIP3P	I	3.5	6.3	6.3	11.4 (11.4)
	II	3.4	6.3	6.4	11.5 (11.4)
	III	3.4	6.2	6.4	11.6 (11.5)
	IV	3.5	6.2	6.2	11.2 (11.3)
	V	3.4	6.3	6.3	11.4 (11.4)
SPC/E	I	3.5	8.3	6.2	13.5 (13.6)
	II	3.5	8.3	6.5	13.7 (13.5)
	III	3.5	8.3	6.0	13.2 (13.6)
	IV	3.5	8.3	6.5	13.5 (13.3)
	V	3.5	8.2	6.3	13.4 (13.4)
TIP4P-D	I	3.3	4.0	-	- (11.6)
	II	3.3	4.0	6.4	11.5 (11.4)
	III	3.3	4.0	6.1	11.2 (11.5)
	IV	3.3	4.0	6.4	11.7 (11.6)
	V	3.4	4.0	6.4	11.5 (11.4)
OPC	I	3.3	4.8	6.1	11.4 (11.6)
	II	3.3	4.9	6.4	11.7 (11.5)
	III	3.3	4.8	6.5	11.9 (11.7)
	IV	3.3	4.8	6.2	11.8 (11.6)
	V	3.3	4.8	6.0	11.5 (11.5)

a The number of ions is calculated from RNA-K^+^ pair distribution function. The numbers in parentheses correspond to a 6.3 Å distance from RNA. A dash means that the radial distribution function did not contain the second minimum and only the values in parentheses are shown.

### Influence of the Number of Ions on the A-RNA Structure

Detailed observations obtained from RDF analyses spurred further investigation into the effect of the number of counterions around the nucleic acid structure. The idea was motivated by the search for the minimum number of counterions that are necessary to stabilize the nucleic acid structures. Therefore, we carried out a series of simulations of the CG duplex using small boxes (S-box) with a variable number of ions ranging from 0 to 18 (corresponding to the net-neutral condition with ∼0.3 M K^+^ concentration). Our previous studies^59,61,64^ have shown that the choice of an explicit water model has a certain effect on the helical parameters of the canonical A-form RNA. However, this effect has always been studied with counterions, which may mask the net effect of the water model.

**Figure 3:**
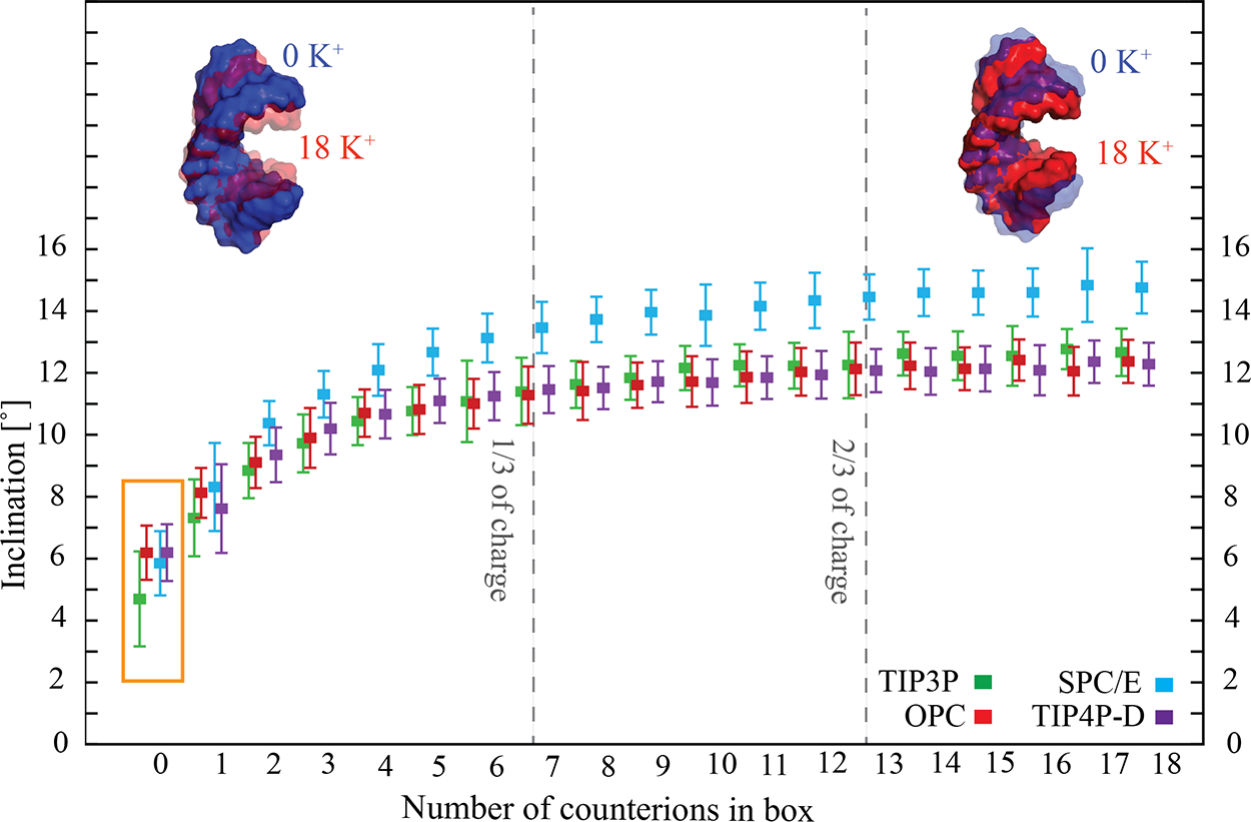
Mean inclination values (and standard deviations represented by error bars) of the CG duplex calculated over complete simulations with different numbers of counterions in the box. The first two base pairs from each end were excluded to avoid the end effects. The gray vertical lines indicate the balance of 1/3 and 2/3 of the charge of the RNA duplex, respectively. The orange rectangle highlights the simulations without any ions. The blue and red 3D structures show how the CG duplex shape differs in the complete absence of explicit ions (blue) and in the presence of 18 K^+^ ions (red).

A complete absence of ions led to a significant reduction in the inclination. With 0 ions, the TIP3P water model gave a visibly lower averaged inclination value compared to the other water models (Figure 3, the orange box). Upon adding K^+^ counterions, the inclination started to increase (i.e., the duplex started to become more compact) and the SPC/E model appeared to have a larger inclination compared to the other three water models. The distinct behavior of the SPC/E water became fully visible from ∼1/3 of net neutral ions present in the system (see Figure 3). The results indicated that the used explicit water model had an effect on the A-RNA structure, but this effect was related to the number of the used counterions.

Upon approaching the net-neutral condition, the inclination became essentially insensitive to the ion concentration. The sufficient number of counterions for the compaction of the A-RNA corresponded to full occupation of the first and second solvation shells, i.e., compensation of 2/3 of the charge of RNA. In other words, the addition of six K^+^ ions (compensating 1/3 of charge of the RNA) led to a significant increase in the A-RNA inclination, while the addition of another six K^+^ ions (12 in total, compensating for 2/3 of the charge of the RNA) led to an essentially saturated inclination value (Figure 3). Subsequent addition of ions caused almost no change in the A-RNA compactness (see Figure 3). These findings are consistent with Manning’s counterion condensation theory,^27−31^ which suggests that an excess of 0.7–0.8 monovalent cations is bound per a phosphate unit and that counterions are found to accumulate near a wire with constant charge density.

**Figure 4:**
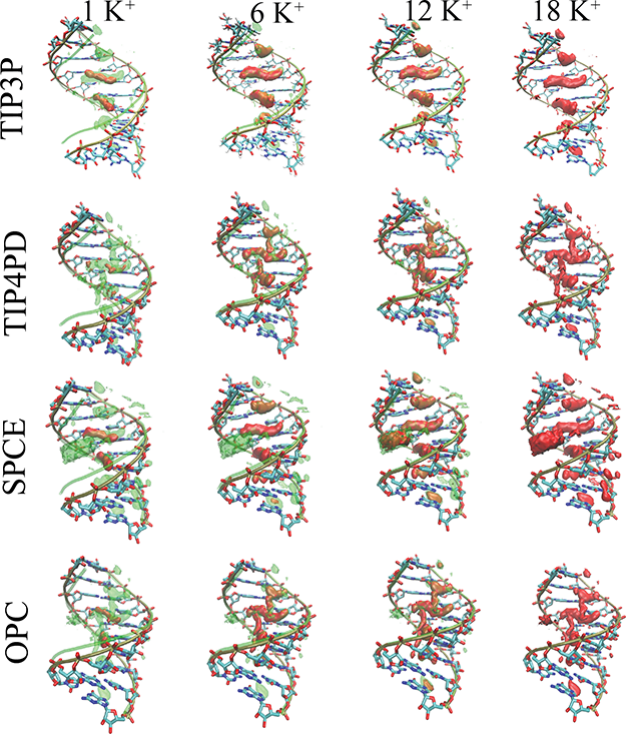
3D density maps of ions calculated from MD simulations of the S-box with different water models and varying numbers of potassium counterions (in red). The total number of counterions used in the MD simulation is given at the top of the figure. The green shadows belong to the reference simulation with 18 ions (net-neutral condition) in the corresponding water model. For ease comparison, all density maps were generated using the same isocontour settings.

In our previous work, we suggested that both the sequence and choice of an explicit water model affect the helical parameters of A-RNA via the modulation of the specific minor groove hydration pattern.^64^ This hydration site between the 2′-OH groups influenced the distance between the 2′-OH groups and thus affected the helical parameters. The different numbers of ions in the first two solvation layers for each water model identified here indicate that the effect of minor-groove hydration was not the only factor affecting the inclination. We further investigated the behavior of the ions around the CG A-RNA duplex in order to identify other possible contributions affecting the behavior of the helical parameters. To describe the ion distribution in detail, we calculated the density maps of potassium ions around the A-RNA structure for all simulations with a varying number of counterions (Figure 4). These maps show that the cations penetrated into minor and major grooves and formed a well-defined interaction pattern. The counterions formed water-mediated contacts and less frequently direct contacts with the electronegative groups. The overall shape of the distribution of ions did not change significantly when the number of the used counterions was increased (Figure 4). In addition, the basic (minimal) density motif that was clearly visible in the simulations with the TIP3P model was repeated in all water models (Figure 4). Furthermore, the cations shared occupancies of these sites with water molecules. Overall, the three-point and four-point water models showed almost similar ion distributions. The TIP3P model showed a very clear localized ion density in the major groove between the base pair steps, and this motif prevailed in the other tested water models (albeit being more “noisy”). In addition, in the four-point models simulations, these densities were vertically connected. The local concentration of ions around the RNA was higher in the SPC/E simulations (see Figure 2 and Table 1), while the 3D density maps showed the same shape. In addition, the second layer of ions connecting the negatively charged backbone atoms of the major groove was also observed in the SPC/E simulations. This higher density was also partially present in the simulations with four-point OPC and TIP4P-D water models.

**Figure 5:**
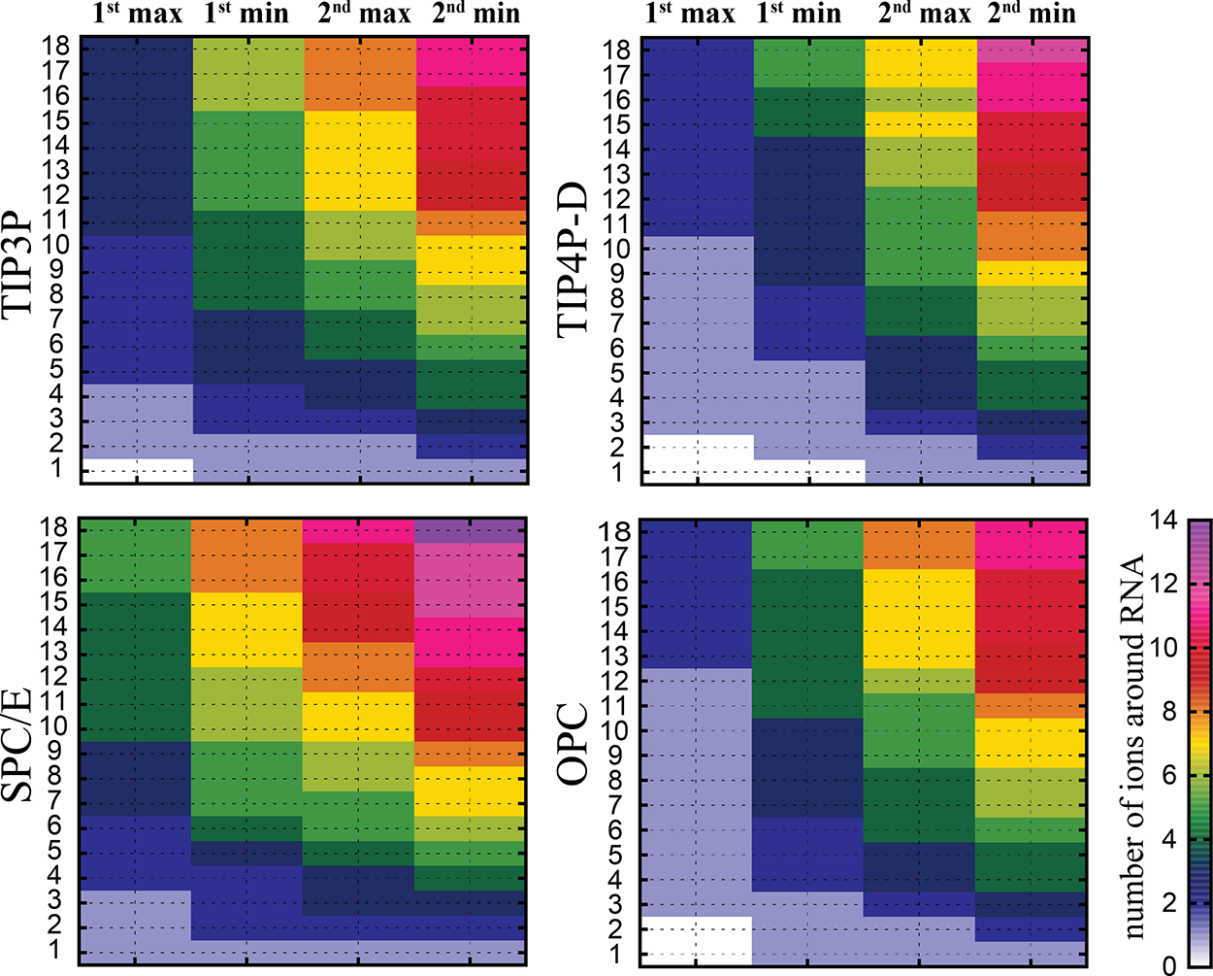
Total number of ions in the first two solvation shells around the CG duplex for different water models with an increasing number of counterions. The *x* axis corresponds to the first and second solvation shells, respectively. The *y* axis corresponds to the number of counterions in the simulation. The color coding represents the number of ions in the respective shells, i.e., the sum of all ions at the corresponding distance from the A-RNA. The color scale ranges from zero (white) to 14 ions (purple). See Table S2 in the Supporting Information for the number of ions in the first and second solvation shells.

Finally, we analyzed the number of ions in the first and second solvation shells around the CG A-RNA duplex in the simulations with a varying number of counterions. We observed that simulations with the SPC/E water led to a higher concentration of ions at the particular distance from the RNA than in the other tested water models (Figure 5). Thus, the SPC/E water model differed significantly from the TIP3P, OPC, and TIP4P-D water models, which behaved similarly. The difference became already apparent when a single K^+^ counterion was added. (Figure 5).

**Figure 6:**
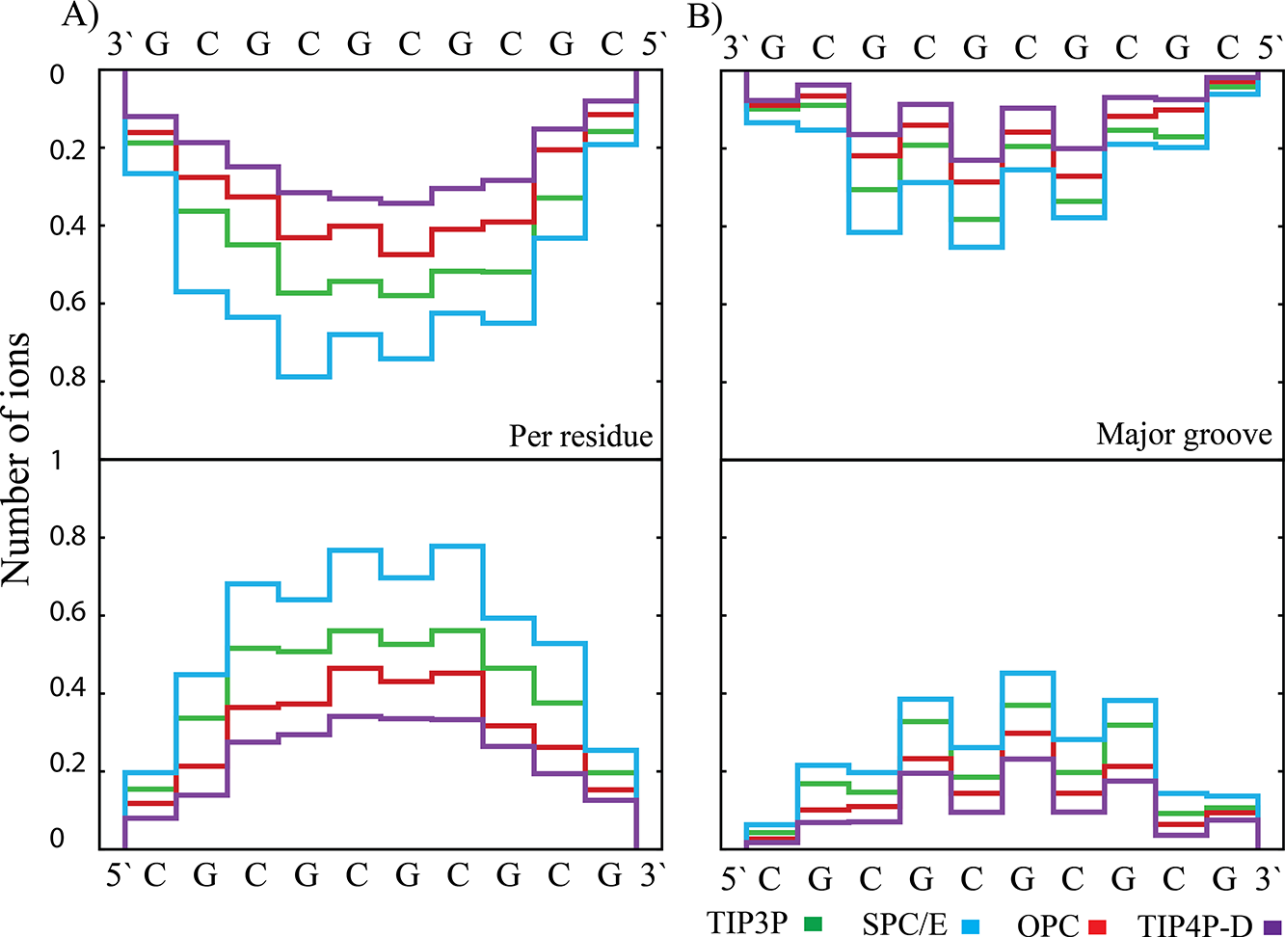
Ion occupancy of each residue in the sequence of the CG duplex in net-neutral simulations using the small box (total number of ions in box was 18 K^+^). The lower part corresponds to the residues from the 5′- to 3′- ends (from left to right), while the upper part describes the residues from the other strand in a reverse order. The average number of ions (A) close to the nucleotide (left) and (B) in the major groove (right). The occupancies were calculated within a distance of 3.5 Å from the center of the nucleotide and the RNA atoms in the major groove, respectively. This distance corresponds to the ions located within the first solvation sphere. The corresponding standard deviations are given in Tables S4 and S5.

The different behavior of the SPC/E water was also evident when we compared the behavior of the ions around (i) the C and G nucleotides, (ii) the atoms in the major and minor grooves, and (iii) the phosphate atoms (see Figure 6 and Figure S1). The average number of ions changed with nucleotides and was symmetric for the two strands. This symmetry was given by the formation of bridges between the GC/CG base pair steps. In addition, we found that the ions mainly bound to the major grooves and to the oxygens of the phosphates, with only a small fraction of ions bound in the minor groove (Figure 6 and Figure S1). In summary, simulations with the SPC/E water model showed a higher number (populations) of bound ions in all binding sites than the simulations with the other tested models.

However, the data above do not show whether the effect was due to the water model or the ion parametrization. To find out why the simulations using the SPC/E water model behaved differently, we prepared CG duplex simulations by swapping the ion parameters for the TIP3P and SPC/E water models. These simulations showed that when the ion parameters were used for the TIP3P water in the simulations with the SPC/E water model, there was a significant decrease in the inclination value (from 14.76° to 13.69°). Conversely, when the ion parameters were used for the SPC/E water model in the simulations with TIP3P, there was a slight increase in the inclination value (from 13.67° to 14.17°). We also analyzed the number of ions in the first and second solvation spheres. The ion exchange in the TIP3P water model led to an increase in the number of ions in both solvation spheres (from 6 to 8 for the first layer and from 11 to 12 for the second layer) and, conversely, in the SPC/E water model, the ion exchange led to a decrease in the number of ions (from 8 to 7 for the first layer and from 14 to 13 for the second layer). Therefore, the different behavior of the SPC/E water model simulations can be mainly explained by the parametrization of the ions, which affected the binding of the ions into the first and second solvation layer.

In conclusion, our new data indicate that the sensitivity of the A-RNA compactness to the water model is a more complex phenomenon than previously thought. The effect apparently reflects an intricate interplay between the water model, the specific ion parametrization, and obviously also the non-bonded terms of the RNA force field, meaning that it is not determined merely by the water model.

### Effect of the Box Size and Ion Concentration on the RNA Structure

We further analyzed the effect of the box size and ion concentration. Besides the net-neutral S-box simulation with a concentration of 0.3 M K^+^, we simulated an M-box with varying ion concentrations from 0.15 M K^+^ to 7.0 M KCl and an L-box with concentrations from 0.02 M K^+^ to 1.2 M KCl (for more information see Methods). The lowest concentration always corresponded to the net-neutral conditions.

**Figure 7:**
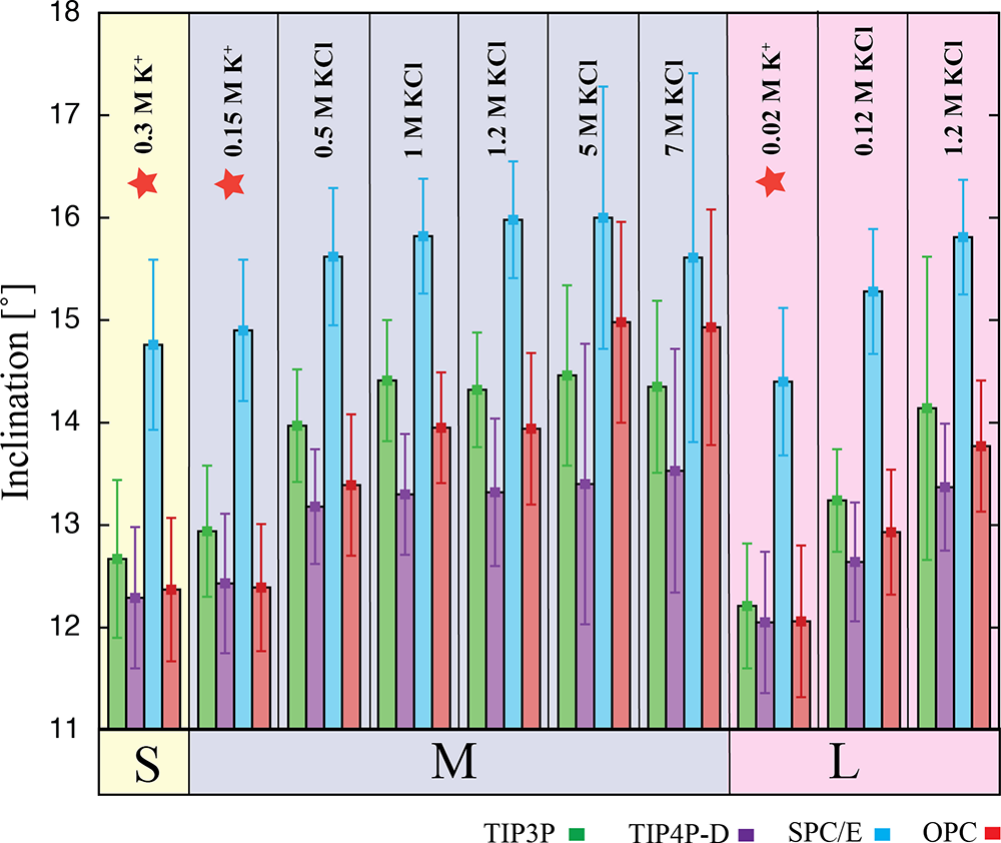
Mean inclination values (for six internal base pairs) of the CG duplex calculated for simulations with a varying box size and salt concentration. Individual boxes are highlighted as follows: S-box in yellow, M-box in gray, and L-box in pink. The orange star indicates simulations with net-neutral conditions.

The results of the net-neutral simulations showed that an increase in the size of the simulation box did not lead to a dramatic change in the inclination (see Figure 7). The simulations of the CG duplex in the S and M-box showed almost the same values of the inclination. For the simulations labeled L, there was a small decrease in the inclination, but even in that case, the largest change in the inclination value was only 0.7 °, which was for the TIP3P simulations. This corroborated the efficient counterion accumulation around the RNA.

A change in the ionic condition from K^+^ to KCl led to a certain increase in the inclination value. This increase was in some cases more than 1° and was evident in both the M-box and L-box (Figure 7). For simulations using the M-box, there was no further significant increase in the inclination value with increasing concentrations.

As mentioned above, the inclination values for the net-neutral simulations did not change dramatically. However, this was not the case for the number of ions in the first and second solvation spheres. The largest number of ions was observed in the net-neutral S-box simulations, and the number of ions decreased when the box size increased (see Table 2 and Table S6).

**Table 2:** Number of Potassium Ions in the First and Second Solvation Shell for Simulations with Different Box Sizes and Concentration of Ions^a^

		first shell		second shell	
box	water	(Å)	N	(Å)	N
S_0.3 M K_^+^	TIP3P	3.5	6.3	6.3	11.4 (11.4)
	SPC/E	3.5	8.3	6.2	13.5 (13.6)
	TIP4P-D	3.3	4.0	6.4	11.5 (11.4)
	OPC	3.3	4.8	6.1	11.4 (11.6)
M_0.15 M K_^+^	TIP3P	3.4	5.3	6.5	9.6 (9.4)
	SPC/E	3.5	7.4		- (11.6)
	TIP4P-D	3.4	3.7		- (9.9)
	OPC	3.3	4.2	6.0	9.4 (9.7)
M_0.5 M KCl_	TIP3P	3.5	9.3	6.2	18.3 (18.5)
	SPC/E	3.5	11.9	6.3	20.9 (20.9)
	TIP4P-D	3.4	5.7	6.0	16.5 (16.5)
	OPC	3.3	7.1	6.2	18.1 (18.3)
M_1.0 M KCl_	TIP3P	3.5	11.9	6.0	23.5 (24.5)
	SPC/E	3.5	14.7	6.4	27.3 (27.0)
	TIP4P-D	3.5	7.4	6.1	22.0 (22.8)
	OPC	3.4	9.2	6.4	23.7 (24.0)
M_1.2 M KCl_	TIP3P	3.5	12.8	6.3	26.8 (26.8)
	SPC/E	3.5	15.7	6.5	29.8 (29.1)
	TIP4P-D	3.4	7.6	6.0	23.3 (24.7)
	OPC	3.3	9.6	6.1	24.9 (26.2)
M_5.0 M KCl_	TIP3P	3.5	26.8	6.0	57.9 (61.8)
	SPC/E	3.5	32.9	6.0	61.6 (65.2)
	TIP4P-D	3.4	16.8	6.0	54.3 (58.5)
	OPC	3.4	23.2	5.9	55.4 (60.3)
M_7.0 M KCl_	TIP3P	3.5	30.7	6.2	70.6 (72.3)
	SPC/E	3.5	39.7	5.9	72.0 (78.1)
	TIP4P-D	3.4	20.6	5.9	64.2 (71.4)
	OPC	3.4	28.0	6.0	66.1 (70.6)
L_0.02 M K_+	TIP3P	3.4	2.2	6.2	6.4 (6.4)
	SPC/E	3.5	3.7		- (8.7)
	TIP4P-D	3.3	1.7		- (7.7)
	OPC	3.3	1.5	6.2	7.1 (7.1)
L_0.12 M KCl_	TIP3P	3.5	3.4	6.5	11.3 (11.1)
	SPC/E	3.5	5.2		- (13.8)
	TIP4P-D	3.3	2.2	6.4	11.7 (11.5)
	OPC	3.3	2.1	6.1	11.3 (11.6)
L_1.2 M KCl_	TIP3P	3.5	6.5	6.2	25.3 (25.7)
	SPC/E	3.5	8.9	6.2	28.1 (28.5)
	TIP4P-D	3.5	3.8	6.0	22.3 (23.6)
	OPC	3.4	3.8	6.0	23.5 (24.7)

a The number of ions is calculated from RNA-K^+^ pair distribution function. The numbers in parentheses correspond to 6.3 Å distance from RNA. A dash means that the radial distribution function did not contain the second minimum and only the values in parentheses are shown.

Regardless of the ion concentrations, the behavior of the radial distribution function showed the same trends for the different water models (see Figures S2–S4). As the concentration increased, the ion concentration in the immediate vicinity of the RNA molecule increased and the individual peaks became more structured. This was also seen in the number of cations in the second solvation layer. At lower concentrations, the second solvation layer was not well defined (Table 2 and Table S6). Indeed, these findings were not unexpected. However, a closer comparison of the radial distribution function showed that at lower concentrations (up to 1.0 M), the radial distribution function of Cl^–^ was not well structured. For concentrations of 1.0 M and above, this function contained three peaks, which became more clearly defined as the concentration increased. These peaks showed a high degree of structuring for simulations below 5.0 and 7.0 M KCl. The positions of the chloride distribution maxima were correlated with the distribution of the potassium ions. The first and second peaks for anions corresponded to the interactions with counterions in the first solvation shell. Further, the first peak also corresponded to the anions directly bound to the electropositive edges of the nucleotides.

### Simulations with Na^+^

In the previous sections, we focused on K^+^ and KCl environments. To compare the effect of the used ions (i.e., Na^+^ vs K^+^ difference) on the A-RNA shape, we performed a series of simulations of the experimentally determined structure (1RNA), which was essentially the AU-tract A-RNA helix and CG duplex. Moreover, the 1RNA simulations in the presence of K^+^ were also used for the comparison with the GC-tract duplex data.

**Figure 8:**
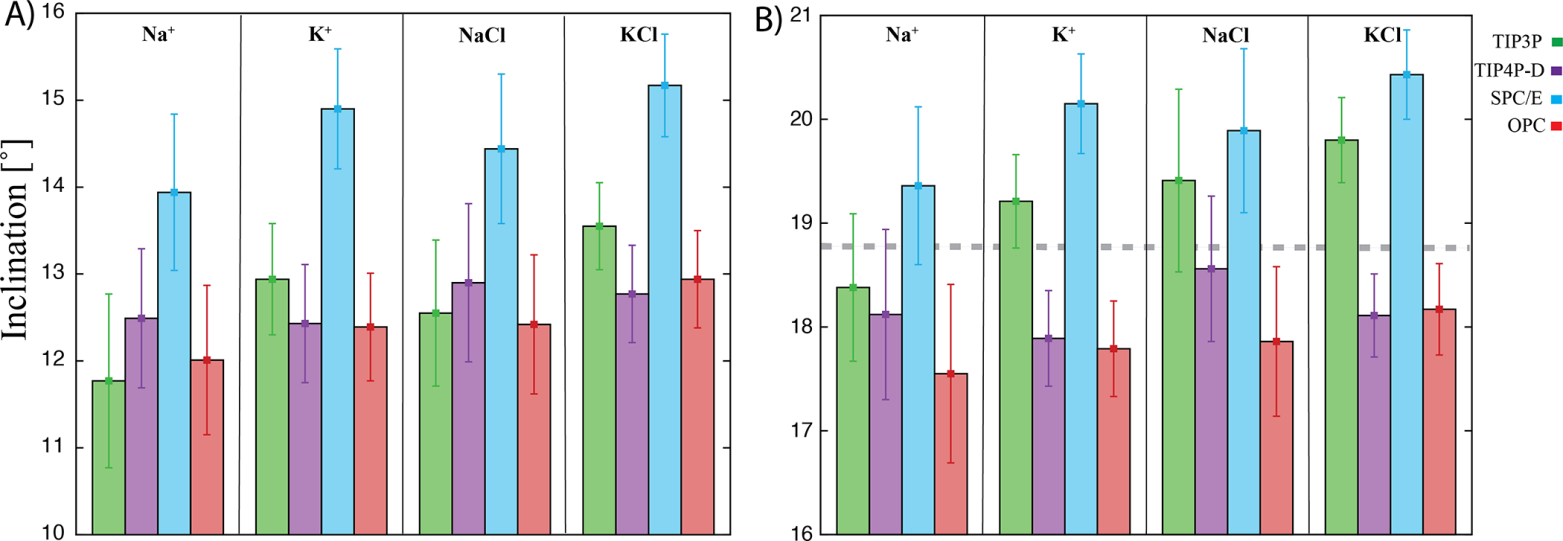
Mean inclination values (and standard deviations represented by error bars). (A) CG duplex and (B) 1RNA r[U(UA)_6_A] duplex calculated over complete M-box simulations using different setup of counterions. The concentrations of ions were as follows: 0.15 M Na^+^/K^+^ and 0.15 M NaCl/KCl, respectively. The number of positively charged ions was 18 (Na^+^/K^+^) or 36 (NaCl/KCl) for the CG duplex simulations and 26 (Na^+^/K^+^) or 52 (NaCl/KCl) for the 1RNA simulations. The first two base pairs from each end were excluded from the analysis to prevent end effects. The gray line represents the inclination value calculated from the crystal structure (18.75°).

The AU-tract simulations showed the same trends as the CG-tract simulations with the K^+^ ions (Figure 8). The range of inclination values in different conditions was from ∼17.5° to 20.5°, which was in good agreement with the experimental (X-ray crystallography) value of 18.75° (for other helical parameters, see Tables S7–S11). In general, the SPC/E water model yielded the largest inclination value, while the change from the net-neutral to the excess salt environment also led to a more compact structure, i.e., an increase in the inclination. For all water models, except the TIP4P-D, there was an increase in the inclination when Na^+^ was changed to K^+^ (see Figure 8). This increase was most pronounced for the TIP3P and SPC/E water models and was larger than the change from net-neutral to salt excess. The simulations with the TIP4P-D water model showed different sensitivity to the Na^+^ and to K^+^ ions. There was a decrease in the inclination when changed from Na^+^ to K^+^, but the simulations behaved similarly to the other water models when there was a change from net-neutral to salt excess ionic conditions. Overall, the data support the abovementioned suggestion that the inclination is not influenced by the water model alone but by a more complex interplay between the water, ion, and RNA parameters. It should be noted that since ion parameters are generally ambiguous, the Na^+^ vs K^+^ difference must always be interpreted as an analysis of two specific sets of ion parameters, meaning that different (but still valid) Na^+^ and K^+^ parameters could result in different Na^+^ vs K^+^ behavior.

### Effect of the RNA Force Field

As a final set of calculations, we compared the A-RNA inclination predicted by the OL3 force field (OPC water model) with three different RNA parameters proposed over the last decade (for other helical parameters, see Tables S12–14). For comparison, we used the 1QC0 and 1RNA duplexes using 0.15 M KCl. Specifically, we used the following force fields: the version by Aytenfisu et al.^85^ (ROC *ff*) in combination with the TIP3P water model, the Chen–Garcia force field^86^ with the TIP3P water model, and the DESRES^89^ force field (DESRES *ff*) in combination with the TIP4P-D water model. It should be noted that the force fields were tested with ion parameters as specified in the Methods section. As some force fields required water/ion models, the comparison results may be affected by different water models since the experiment/test was not possible to conduct under a uniform set of conditions.

**Figure 9:**
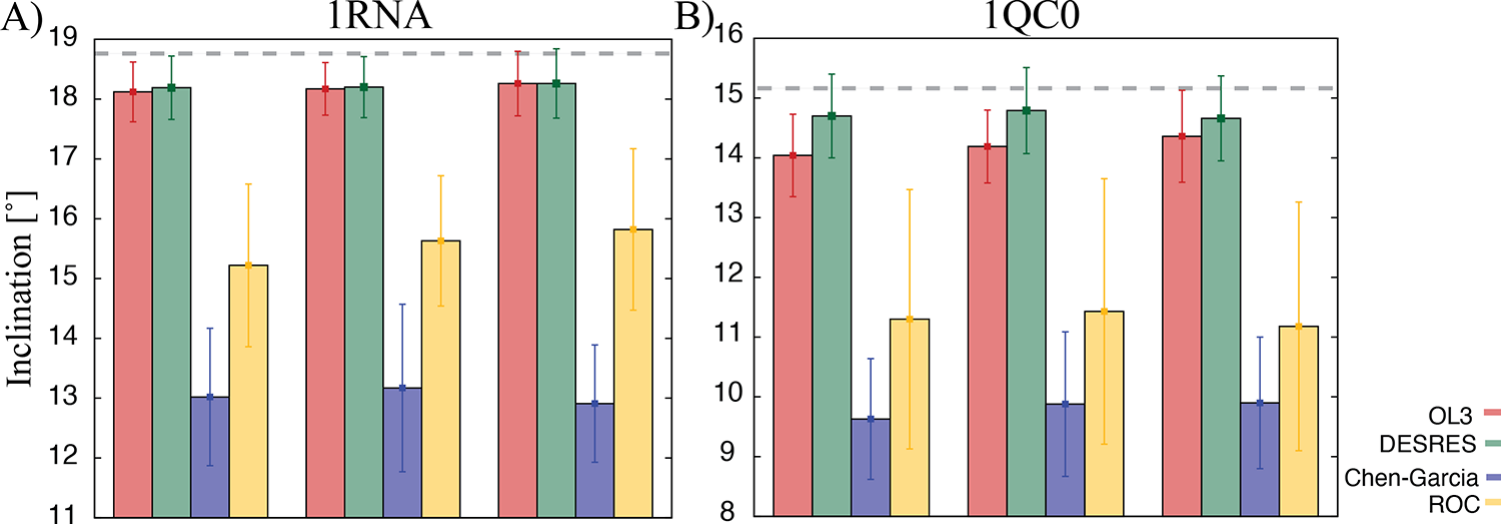
Mean inclination values (and standard deviations represented by error bars) of (A) 1RNA and (B) 1QC0 duplexes were calculated on the basis of three completed independent simulations using different solute force fields. The first two base pairs from each were excluded to prevent end effects. The gray line represents inclination calculated from the crystal structure of 1RNA (18.75°) and 1QC0 (15.15°), respectively.

For the 1QC0 and 1RNA duplexes, the best agreement with the experimental values of the inclination was shown by DESRES^89^ and OL3^67−70^ force fields (see Figure 9 and Tables S12 and S13). Both force fields described these two canonical A-RNAs very well. This result is in agreement with the original work^89^ and is consistent with the fact that the DESRES *ff* was tuned to give good performance for the A-form RNA (both duplex and single strand). The DESRES *ff* is known to outperform the OL3 force field (unless the latter is combined with the gHBfix) for RNA tetranucleotides. However, previous research showed that the DESRES *ff* had major problems describing some folded RNAs,^75^ which was not apparent in the original publication. In fact, simulations of the kink-turn RNA motif and the ribosomal L1-stalk RNA segment using DESRES *ff* led to complete rearrangement or disruption of the experimental structures.^75^ The OL3 force field dealt with the description of these systems without significant problems.^75,95−97^ The remaining two force fields, i.e., Chen–Garcia *ff* and ROC *ff* appeared to underestimate the inclination value, meaning that they may underrate the compactness of dsRNA (see Tables S12 and S13). The underestimation of the inclination value for the ROC *ff* was already apparent in the original paper,^85^ where the [U(AU)_6_A]_2_ duplex was studied. We also note that the ROC *ffs* may show an increased tendency for duplex end fraying (see Tables S12 and S13). However, a converged analysis of fraying would require a significantly longer simulation time scale, so we only present the data in the Supporting Information. The comparison of the inclination between the OL3 force field and the preceding AMBER ff99bsc0 version can be found in ref (98), showing mutual agreement. Note that the ff99bsc0 was not included in our study because it is prone to the formation of ladder-like RNA structures.^69^

Although the crystal structures provided direct information about the inclination and the A-RNA duplex compactness via the observed electron densities, the RNA structure might have been affected by the crystalline conditions. Besides the crystal packing, also the local ionic and water environment around the RNA molecules did not fully resemble the solvent conditions. To complement our force field test, we performed simulations of the solution NMR structure of the 2GBH duplex and compared them with the predicted and experimentally observed NOEs and with the inclination values obtained from the simulations and the refined NMR structure. We observed that the agreement with experimental NOEs in the simulations did not implicate good congruence with the inclination value observed in the refined NMR structure. This might indicate that the inclination as observed in the refined NMR structures might have also been affected by the refinement protocol and therefore cannot be used for direct comparison of the force field performance (see Tables S14 and S15 and Figure S5).

## Conclusions

The ionic conditions are a key factor in the RNA solution experiments, so it is important to fully understand the setting of the ionic conditions in the simulations and its effect on the predicted RNA structural dynamics. We have performed an extensive series of simulations (more than 250 simulations with total simulation time of over 250 μs) to investigate the effect of the solvation and co-solvation conditions with respect to not only the type of ions but also the water model, ionic concentration, box size, and choice of the force field on the compactness of the canonical A-RNA double helix as the most abundant RNA motif.

The helical structure of A-form RNA duplexes was dominantly influenced by the solute force field, when moderately modulated by ionic parameters and concentration, and by the water model. On the other hand, the structure of A-form helices was rather invariant to the box size, even when the solvation box contained only negligible part of the solvent bulk outside the hydration spheres around the RNA. While simulations with small boxes have been considered for a long time to provide insufficient description of realistic ionic conditions, our data show that this simulation setting can be satisfactory as well. Among the tested solute force fields, the OL3 and DESRES described correctly the A-form duplex, whereas the ROC and Chen–Garcia force fields had a tendency to underestimate the inclination and the roll helical parameters, which affected the shape of the duplex. For this comparison, we used the experimental inclination value obtained under crystalline conditions, which might have been affected by crystal packing. Nonetheless, the comparison with solvent experiments was not straightforward as they did not provide direct information about the inclination and the A-form duplex compactness.

It has been suggested in the past that the effect of the water model on the A-RNA compactness is due to a specific hydration pattern in the A-RNA minor groove. The present simulations suggest a more complex picture, showing that the compactness of the A-RNA duplexes was influenced by the interplay between the water model, ion parameters and conditions, and the non-bonded terms of the RNA force field. Thus, while the water models affected the compactness of the A-RNA duplex, the differences between the different water models were far from being uniform.

## Data Availability

The starting structures and NMR data were obtained from a PDB bank (RCSB PDB: https:/www.rcsb.org) or prepared using Nucleic Acid Builder of AmberTools (http://ambermd.org). All molecular dynamics were carried out in Amber16 (http://ambermd.org). The simulations were analyzed using a *cpptraj* module (AMBER tool, http://ambermd.org). The simulations were visualized using a VMD program (http://www.ks.uiuc.edu/Research/vmd) or PyMOL program (https://pymol.org). The radial distribution function and identification and classification of the type of base pair family were calculated using an in-house programs, which are available in the SI (RDF_ions.zip and BPandStacks_SI.zip). All graphs were prepared using Gnuplot (http://www.gnuplot.info).

## Supporting Information Available

In-house programs for calculation of the radial distribution function and identification and classification of the type of base pair family (ZIP, ZIP)
Description of simulation setup; list of all performed simulations; RNA-K^+^ pair distribution function for all simulations of CG duplex; mean values and standard deviation of end-to-end distances of the CG duplex; mean values of average number of ions (and standard deviations) close to each nucleotide in the sequence of the CG duplex; mean values of average number of ions (and standard deviations) in the major groove of the CG duplex; mean values of the helical parameters (and standard deviations represented by error bars) of studied 1RNA calculated over complete multiple simulations using different setup of counterions; mean values of the helical parameters (and standard deviations represented by error bars) of studied 1RNA calculated over complete multiple simulations using different force fields; mean values of helical parameters (and standard deviations) of the CG duplex calculated over complete multiple simulations using different setup of counterions; mean values of the helical parameters (and standard deviations represented by error bars) of studied 1QC0 calculated over complete multiple simulations using different force fields; mean values of the helical parameters (and standard deviations represented by error bars) of studied 2GBH calculated over complete multiple simulations using different force fields; χ_NOE_^2^ values of nuclear Overhauser effect intensities calculated over complete multiple simulations of the 2GBH duplex using different force fields; ion occupancy of each residue in the sequence of artificial CG duplex in net-neutral simulations using small box; graphs of radial concentration profiles of ions calculated for all simulations of CG duplex; correlation between the total χ_NOE_^2^ values and the difference between the inclination values obtained from the 2GBH simulations (using different force fields) and the experimental inclination value (PDF)
