# Correction to “Sensitivity of the RNA Structure to Ion Conditions as Probed by Molecular Dynamics Simulations of Common Canonical RNA Duplexes”

**DOI:** 10.1021/acs.jcim.4c01067

**Published:** 2024-06-27

**Authors:** Petra Kührová, Vojtěch Mlýnský, Michal Otyepka, Jiří Šponer, Pavel Banáš

This erratum is to correct Table S15 and Figure S5 published in the Supporting Information of our recent article. Both Table S15 and Figure S5 have been updated with the correct χ^2^*NOE* values of nuclear Overhauser effect intensities. We emphasize that this correction does not affect any of the results or conclusions presented in the main text of the article.

**Table S15:** χ_*NOE*_^2^ Values of Nuclear Overhauser Effect Intensities Calculated over Complete Multiple Simulations of 2GBH Duplex Using Different Force Fields

	χ^2^		
Force field	Sim #1	Sim #2	Sim #3
DESRES	0.241	0.254	0.245
Chen-Garcia	0.245	0.244	0.249
ROC	0.322	0.304	0.306
OL3	0.250	0.252	0.254

**Figure S5:**
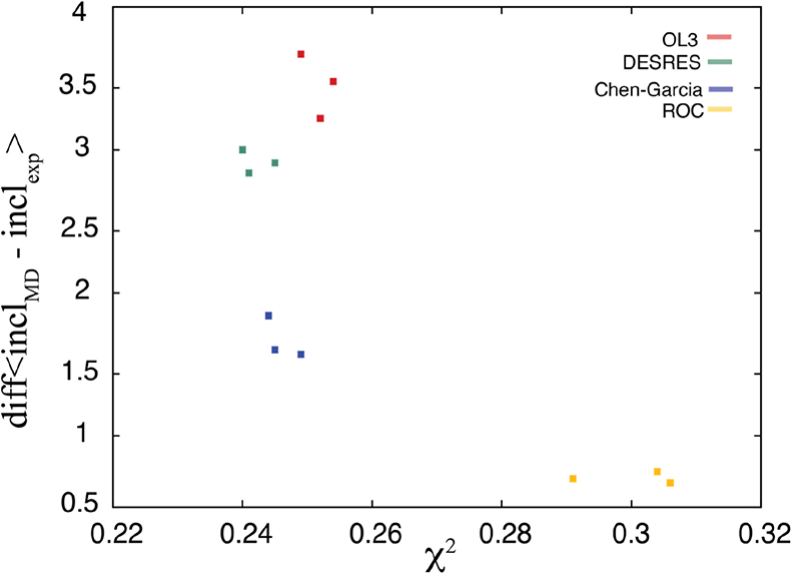
Correlation between the total χ_*NOE*_^2^ values and the difference between the inclination values obtained from the 2GBH simulations (using different force fields) and the experimental inclination value. The experimental inclination value was calculated as the average value over all NMR frames.

To avoid misleading readers about the χ^2^*NOE* values, we provide the corrected Table S15 and Figure S5.

