# Supplementary material for: Sensitivity of the RNA Structure to Ion Conditions as Probed by Molecular Dynamics Simulations of Common Canonical RNA Duplexes
Source: J Chem Inf Model. 2023 Mar 29;63(7):2133–46. doi: 10.1021/acs.jcim.2c01438 (PMC10091408; doi:10.1021/acs.jcim.2c01438)
Supplement: Supplementary file 3 — ci2c01438_si_003.pdf [file ci2c01438_si_003.pdf]

Supporting Information for:

Sensitivity of RNA Structure to Ion Conditions as probed by MD Simulations of Common Canonical RNA duplexes

Petra Kührová, Vojtěch Mlýnský, Jiří Šponer, Michal Otyepka and Pavel Banáš

Simulation setup

Each RNA-solvent system was minimized, by first optimizing the waters and ions, while the positions of the RNA molecule remained constrained. Subsequently, all RNA atoms were frozen and the solvent molecules with counter-ions were allowed to move during a 1000-step minimization, followed by 500–ps–long MD runs under [NpT] conditions (p = 1 atm., T = 298.16 K) in order to relax the total density. After this, the solute and nucleobases were relaxed by several minimization runs, with decreasing force constants applied to the sugar-phosphate backbone atoms. After the relaxation, the system was heated in two steps: the first step involved heating in NVT condition (for 100 ps) and then the system is equilibrating in NpT condition for additional 100 ps. The particle-mesh Ewald (PME) method for treating electrostatic interactions was used, and all simulations were performed under periodic boundary conditions in the [NpT] ensemble at 298.16 K using weak-coupling Berendsen thermostat (*Berendsen, H.; 1984; JChemPhys 81 (8) 3684; doi.org/10.1063/1.448118*) with coupling time of 1 ps. The SHAKE algorithm, with a tolerance of 10^{-5} Å, was used to fix the positions of all hydrogen atoms, and a 10.0 Å cut-off was applied to non-bonding interactions to allow a 2-fs integration step.

Supporting Tables

Table S1: List of all performed simulations.

System	Solute force field	Box size	Water model	Ions	Targeted concentration	# of waters	# of ions	Number of simulations
CG	OLO3	S	TIP3P<br>TIP4P-D<br>SPC/E<br>OPC	K^+	0 – 0.3 M	3205	0-18 K^+	1x 0-0.29 M<br>5x 0.3 M
		M	TIP3P<br>TIP4P-D<br>SPC/E<br>OPC	Na^+	0.15 M	6438	18 Na^+	1x
		M	TIP3P<br>TIP4P-D<br>SPC/E<br>OPC	NaCl	0.15 M	6314	35 Na^+<br>17 Cl^-	1x
		M	TIP3P<br>TIP4P-D<br>SPC/E<br>OPC	K^+	0.15 M	6348	18 K^+	1x
		M	TIP3P<br>TIP4P-D<br>SPC/E<br>OPC	KCl	0.15 M, 0.5 M, 1 M, 1.2 M, 5 M, 7 M	6220-6348	35, 75, 129, 154, 573, 748 K^+<br>17, 57, 111, 136, 555, 730 Cl^-	1x
		L	TIP3P<br>TIP4P-D<br>SPC/E	KCl	0.02 – 1.2 M	43473-44937	18, 120, 933 K^+	1x

			OPC				36, 138, 951 Cl^-	
	OLO3	M	TIP3P	K^+	0.15 M	9591	26 K^+	5x
			TIP4P-D			9617		
			SPC/E			9590		
			OPC			9654		
	OLO3	M	TIP3P	Na^+	0.15 M	9591	26 Na^+	5x
			TIP4P-D			9669		
			SPC/E			9590		
			OPC			9654		
1RNA	OLO3	M	TIP3P	NaCl	0.15 M	9540	52 Na^+ 26 Cl^-	5x
			TIP4P-D			9617		
			SPC/E			9539		
			OPC			9654		
	OLO3	M	TIP3P	KCl	0.15 M	9541	52 K^+ 26 Cl^-	5x
			TIP4P-D			9617		
			SPC/E			9540		
			OPC			9654		
	DESRES	M	TIP4P-D	KCl	0.15 M	9619	52 K^+ 26 Cl^-	3x
	Chen-Garcia	M	TIP3P			9541		
	ROC	M	TIP3P			9542		
1QC0	OLO3	M	OPC	KCl	0.15 M	6433	36 K^+ 18 Cl^-	3x
	DESRES		TIP4P-D			6433		
	Chen-Garcia		TIP3P			6584		
	ROC		TIP3P			6321		
2GBH	OLO3	M	OPC	KCl	0.15 M	5852	30 K^+	3x

DESRES	TIP4P-D		5896	16 Cl^-
Chen-Garcia	TIP3P		5922	
ROC	TIP3P		5938	

Table S2: Number of ions (N) in the first and second solvation shells based on RNA-K^+ pair distribution function for all net-neutral S-box simulations of CG duplex with varying number of ions (#ions). A dash means that the radial distribution function did not contain the second minimum and only the values in parentheses are shown. This value corresponds to 6.3 Å distance from RNA.

water	#ions	first shell		second shell	
		(Å)	N	(Å)	N
TIP3P	1	3.4	0.7	6.0	1.0 (1.0)
	2	3.4	1.3	6.9	2.0 (2.0)
	3	3.4	1.9	6.7	2.9 (2.9)
	4	3.4	2.2	6.5	3.7 (3.6)
	5	3.4	2.6	6.6	4.5 (4.4)
	6	3.4	2.9	6.3	5.0 (5.0)
	7	3.4	3.3	6.4	5.7 (5.7)
	8	3.4	3.6	6.3	6.2 (6.2)
	9	3.5	4.1	6.3	6.9 (6.9)
	10	3.4	4.1	6.3	7.4 (7.4)
	11	3.4	4.4	6.5	8.1 (8.0)
	12	3.4	4.7	6.2	8.4 (8.5)
	13	3.5	4.9	6.3	8.8 (8.8)
	14	3.5	5.2	6.4	9.6 (9.5)
	15	3.4	5.5	6.3	10.0 (10.0)
	16	3.5	5.8	6.5	10.6 (10.4)
	17	3.5	6.1	6.3	11.0 (11.0)
	18	3.5	6.3	6.3	11.4 (11.4)
SPC/E	1	3.4	0.8	6.4	1.1 (1.1)
	2	3.4	1.6	6.5	2.2 (2.1)
	3	3.4	2.2	7.0	3.1 (3.1)

	4	3.5	2.8	6.2	4.0 (4.0)
	5	3.5	3.3	6.4	4.9 (4.9)
	6	3.4	3.8	5.9	5.7 (5.8)
	7	3.5	4.3	6.4	6.6 (6.6)
	8	3.5	4.7	6.0	7.1 (7.2)
	9	3.5	5.2	7.0	8.3 (8.0)
	10	3.5	5.7	7.0	8.7 (8.6)
	11	3.5	6.0	6.3	9.4 (9.4)
	12	3.5	6.3	6.5	10.1 (10.0)
	13	3.5	6.6	6.6	10.8 (10.6)
	14	3.5	7.0	6.4	11.1 (11.0)
	15	3.5	7.3	6.3	11.8 (11.8)
	16	3.5	7.7	6.3	12.5 (12.5)
	17	3.6	8.1	6.3	12.9 (12.9)
	18	3.5	8.3	6.2	13.5 (13.6)
TIP4P-D	1	3.3	0.5	5.9	1.0 (1.0)
	2	3.3	0.8	-	- (2.0)
	3	3.3	1.2	5.9	2.8 (2.9)
	4	3.3	1.4	5.8	3.5 (3.7)
	5	3.3	1.7	5.9	4.3 (4.4)
	6	3.3	1.9	6.0	5.0 (5.1)
	7	3.3	2.1	6.1	5.7 (5.8)
	8	3.3	2.3	-	- (6.4)
	9	3.3	2.5	6.2	7.0 (7.1)
	10	3.3	2.7	6.0	7.4 (7.7)
	11	3.3	2.8	-	- (8.1)

	12	3.3	3.1	6.0	8.4 (8.7)
	13	3.3	3.2	6.0	8.8 (9.1)
	14	3.3	3.4	6.3	9.7 (9.7)
	15	3.3	3.5	6.0	9.8 (10.2)
	16	3.3	3.7	6.1	10.3 (10.6)
	17	3.3	3.8	6.4	11.1 (11.2)
	18	3.3	4.0	-	- (11.6)
OPC	1	3.3	0.6	6.2	1.0 (1.0)
	2	3.2	1.0	5.6	1.9 (2.0)
	3	3.3	1.4	-	- (2.8)
	4	3.3	1.7	5.8	3.5 (3.6)
	5	3.3	2.0	7.0	4.7 (4.4)
	6	3.3	2.3	6.6	5.2 (5.0)
	7	3.3	2.6	-	- (5.8)
	8	3.3	2.8	6.3	6.4 (6.4)
	9	3.3	3.0	6.9	7.4 (6.9)
	10	3.3	3.2	7.7	8.4 (7.5)
	11	3.3	3.5	6.0	7.9 (8.1)
	12	3.3	3.7	6.1	8.4 (8.6)
	13	3.3	4.0	6.4	9.5 (9.4)
	14	3.3	4.0	6.3	9.7 (9.7)
	15	3.3	4.2	7.3	11.3 (10.2)
	16	3.3	4.5	6.2	10.6 (10.8)
	17	3.3	5.0	6.3	11.2 (11.2)
	18	3.3	4.8	6.1	11.4 (11.6)

Table S3: Mean values and standard deviations of end-to end distances of CG duplex calculated over complete multiple net-neutral simulations of S-box.

Water model	End-to-end distance [Å]				
	Sim #1	Sim #2	Sim #3	Sim #4	Sim #5
TIP3P	24.10 ± 1.83	24.30 ± 1.53	24.05 ± 1.77	24.26 ± 1.59	24.19 ± 1.74
SPC/E	23.87 ± 1.78	23.84 ± 1.96	23.24 ± 2.17	23.46 ± 2.05	23.48 ± 1.73
TIP4P-D	24.38 ± 2.39	24.35 ± 2.16	24.89 ± 2.16	25.08 ± 1.90	24.72 ± 2.01
OPC	24.72 ± 2.36	24.20 ± 2.30	24.23 ± 2.32	23.82 ± 2.40	24.49 ± 1.94

Table S4: Mean values of average number of ions (and standard deviations) close to each nucleotide in the sequence of CG duplex in net-neutral simulations using small box.

Residue	Water			
	TIP3P	SPC/E	TIP4P-D	OPC
5’ C_1	0.15 ± 0.38	0.20 ± 0.43	0.08 ± 0.28	0.12 ± 0.34
G_2	0.34 ± 0.57	0.45 ± 0.66	0.14 ± 0.37	0.21 ± 0.46
C_3	0.52 ± 0.67	0.68 ± 0.79	0.28 ± 0.50	0.36 ± 0.57
G_4	0.51 ± 0.64	0.64 ± 0.71	0.29 ± 0.51	0.37 ± 0.57
C_5	0.56 ± 0.67	0.77 ± 0.76	0.34 ± 0.54	0.46 ± 0.62
G_6	0.53 ± 0.62	0.70 ± 0.68	0.34 ± 0.53	0.43 ± 0.59
C_7	0.56 ± 0.66	0.78 ± 0.75	0.33 ± 0.53	0.45 ± 0.61
G_8	0.46 ± 0.61	0.59 ± 0.68	0.26 ± 0.49	0.32 ± 0.52
C_9	0.38 ± 0.58	0.53 ± 0.67	0.19 ± 0.43	0.26 ± 0.49
3’ G_{10}	0.20 ± 0.44	0.25 ± 0.50	0.13 ± 0.35	0.15 ± 0.39
5’ C_{11}	0.16 ± 0.39	0.19 ± 0.43	0.08 ± 0.28	0.12 ± 0.34
G_{12}	0.32 ± 0.56	0.43 ± 0.64	0.15 ± 0.39	0.21 ± 0.45
C_{13}	0.51 ± 0.68	0.65 ± 0.77	0.28 ± 0.51	0.39 ± 0.59
G_{14}	0.52 ± 0.64	0.62 ± 0.70	0.30 ± 0.51	0.41 ± 0.58
C_{15}	0.58 ± 0.67	0.74 ± 0.74	0.34 ± 0.54	0.47 ± 0.63
G_{16}	0.54 ± 0.63	0.68 ± 0.68	0.33 ± 0.52	0.40 ± 0.57
C_{17}	0.57 ± 0.67	0.79 ± 0.76	0.32 ± 0.52	0.43 ± 0.60
G_{18}	0.45 ± 0.60	0.64 ± 0.69	0.25 ± 0.47	0.33 ± 0.53
C_{19}	0.36 ± 0.57	0.57 ± 0.69	0.19 ± 0.42	0.27 ± 0.50
3’ G_{20}	0.19 ± 0.43	0.27 ± 0.51	0.12 ± 0.34	0.16 ± 0.40

Table S5: Mean values of average number of ions (and standard deviations) in the major groove of CG duplex in net-neutral simulations using small box.

Residue	Water			
	TIP3P	SPC/E	TIP4P-D	OPC
5' C_1	0.04 ± 0.20	0.06 ± 0.24	0.02 ± 0.13	0.03 ± 0.16
G_2	0.17 ± 0.37	0.22 ± 0.41	0.07 ± 0.25	0.10 ± 0.30
C_3	0.15 ± 0.36	0.20 ± 0.41	0.07 ± 0.26	0.11 ± 0.31
G_4	0.33 ± 0.48	0.39 ± 0.49	0.20 ± 0.40	0.23 ± 0.43
C_5	0.18 ± 0.40	0.26 ± 0.46	0.09 ± 0.29	0.14 ± 0.36
G_6	0.37 ± 0.49	0.45 ± 0.51	0.23 ± 0.42	0.30 ± 0.46
C_7	0.20 ± 0.41	0.28 ± 0.47	0.10 ± 0.30	0.14 ± 0.36
G_8	0.32 ± 0.47	0.38 ± 0.49	0.17 ± 0.38	0.21 ± 0.41
C_9	0.09 ± 0.29	0.14 ± 0.35	0.04 ± 0.19	0.06 ± 0.25
3' G_{10}	0.11 ± 0.31	0.14 ± 0.35	0.07 ± 0.26	0.09 ± 0.29
5' C_{11}	0.04 ± 0.20	0.06 ± 0.24	0.02 ± 0.13	0.03 ± 0.17
G_{12}	0.17 ± 0.38	0.20 ± 0.40	0.07 ± 0.26	0.10 ± 0.30
C_{13}	0.15 ± 0.37	0.19 ± 0.40	0.07 ± 0.26	0.12 ± 0.33
G_{14}	0.34 ± 0.48	0.38 ± 0.49	0.20 ± 0.40	0.27 ± 0.45
C_{15}	0.19 ± 0.41	0.25 ± 0.45	0.10 ± 0.30	0.16 ± 0.37
G_{16}	0.38 ± 0.49	0.45 ± 0.51	0.23 ± 0.42	0.29 ± 0.46
C_{17}	0.19 ± 0.40	0.29 ± 0.47	0.09 ± 0.28	0.14 ± 0.35
G_{18}	0.31 ± 0.46	0.42 ± 0.50	0.16 ± 0.37	0.22 ± 0.42
C_{19}	0.09 ± 0.29	0.15 ± 0.37	0.04 ± 0.19	0.07 ± 0.25
3' G_{20}	0.10 ± 0.30	0.13 ± 0.34	0.08 ± 0.27	0.09 ± 0.29

Table S6: Number of ions (N) in the first and second solvation shells based on RNA-K^+ pair distribution function for all simulations. The numbers in brackets correspond to 6.3 Å distance from RNA. A dash means that the radial distribution function did not contain the second minimum and only the values in parentheses are shown.

		First shell				Second shell			
		K^+		Cl^-		K^+		Cl^-	
box	water	(Å)	N	(Å)	N	(Å)	N	(Å)	N
$S_{0.3M\ K+}$	TIP3P	3.5	6.3	3.5	0.0	6.3	11.4 (11.4)	6.3	0.0
	SPC/E	3.5	8.3	3.5	0.0	6.2	13.5 (13.6)	6.3	0.0
	TIP4P-D	3.3	4.0	3.5	0.0	6.4	11.5 (11.4)	6.3	0.0
	OPC	3.3	4.8	3.5	0.0	6.1	11.4 (11.6)	6.3	0.0
$M_{0.15M\ K+}$	TIP3P	3.4	5.3	3.5	0.0	6.5	9.6 (9.4)	6.3	0.0
	SPC/E	3.5	7.4	3.5	0.0	-	- (11.6)	6.3	0.0
	TIP4P-D	3.4	3.7	3.5	0.0	-	- (9.9)	6.3	0.0
	OPC	3.3	4.2	3.5	0.0	6.0	9.4 (9.7)	6.3	0.0
$M_{0.5M\ KCl}$	TIP3P	3.5	9.3	3.5	0.3	6.2	18.3 (18.5)	6.3	2.9
	SPC/E	3.5	11.9	3.5	0.2	6.3	20.9 (20.9)	6.3	2.8
	TIP4P-D	3.4	5.7	3.5	0.3	6.0	16.5 (16.5)	6.3	2.7
	OPC	3.3	7.1	3.5	0.2	6.2	18.1 (18.3)	6.3	2.4
$M_{1M\ KCl}$	TIP3P	2.8	11.9	3.5	0.8	6.0	23.5 (24.5)	6.3	6.7
	SPC/E	2.8	14.7	3.5	0.6	6.4	27.3 (27.0)	6.3	6.5
	TIP4P-D	2.8	7.4	3.5	0.8	6.1	22.0 (22.8)	6.3	6.3
	OPC	2.7	9.2	3.5	0.5	6.4	23.7 (24.0)	6.3	5.5
$M_{1.2M\ KCl}$	TIP3P	2.8	12.8	3.5	0.9	6.3	26.8 (26.8)	6.3	8.3
	SPC/E	2.8	15.7	3.5	0.8	6.5	29.8 (29.1)	6.3	8.0
	TIP4P-D	2.8	7.6	3.5	1.0	6.0	23.3 (24.7)	6.3	7.8

	OPC	2.7	9.6	3.5	0.7	6.1	24.9 (26.2)	6.3	7.0
$M_{5M\ KCl}$	TIP3P	2.8	26.8	3.5	6.2	6.0	57.9 (61.8)	6.3	41.6
	SPC/E	2.8	32.9	3.5	6.5	6.0	61.6 (65.2)	6.3	43.8
	TIP4P-D	2.8	16.8	3.5	6.8	6.0	54.3 (58.5)	6.3	40.1
	OPC	2.7	23.2	3.5	5.5	5.9	55.4 (60.3)	6.3	39.3
$M_{7M\ KCl}$	TIP3P	2.8	30.7	3.5	6.2	6.2	70.6 (72.3)	6.3	41.6
	SPC/E	2.8	39.7	3.5	10.0	5.9	72.0 (78.1)	6.3	58.8
	TIP4P-D	2.8	20.6	3.5	9.8	5.9	64.2 (71.4)	6.3	53.4
	OPC	2.7	28.0	3.5	7.9	6.0	66.1 (70.6)	6.3	51.3
$L_{0.02M\ K+}$	TIP3P	2.8	2.2	3.5	0.0	6.2	6.4 (6.4)	6.3	0.0
	SPC/E	2.8	3.7	3.5	0.0	-	- (8.7)	6.3	0.0
	TIP4P-D	2.8	1.7	3.5	0.0	-	- (7.7)	6.3	0.0
	OPC	2.7	1.5	3.5	0.0	6.2	7.1 (7.1)	6.3	0.0
$L_{0.12M\ KCl}$	TIP3P	2.8	3.4	3.5	0.0	6.5	11.3 (11.1)	6.3	0.4
	SPC/E	2.8	5.2	3.5	0.0	-	- (13.8)	6.3	0.4
	TIP4P-D	2.8	2.2	3.5	0.0	6.4	11.7 (11.5)	6.3	0.4
	OPC	2.7	2.1	3.5	0.0	6.1	11.3 (11.6)	6.3	0.3
$L_{1.2M\ KCl}$	TIP3P	2.8	6.5	3.5	0.9	6.2	25.3 (25.7)	6.3	7.7
	SPC/E	2.8	8.9	3.5	0.7	6.2	28.1 (28.5)	6.3	7.7
	TIP4P-D	2.8	3.8	3.5	0.9	6.0	22.3 (23.6)	6.3	7.0
	OPC	2.7	3.8	3.5	0.6	60	23.5 (24.7)	6.3	6.4

Table S7: Mean values of inclination (and standard deviations) of 1RNA duplex calculated over complete multiple simulations using different setup of counterions. The first two base pairs from each end terminal of the strands were excluded to prevent end effects. The inclination of crystal structure is 18.75 °.

Water model	Ions	Inclination [°]				
		Sim #1	Sim #2	Sim #3	Sim #4	Sim #5
TIP3P	Na^+	18.64 ± 0.82	18.48 ± 0.82	18.37 ± 0.84	18.27 ± 1.12	18.38 ± 0.71
	K^+	19.14 ± 0.55	19.21 ± 0.45	19.12 ± 0.48	19.22 ± 0.51	19.12 ± 0.46
	NaCl	19.46 ± 1.15	19.85 ± 1.17	19.41 ± 0.88	19.23 ± 0.96	19.18 ± 0.89
	KCl	19.93 ± 0.50	19.75 ± 0.45	19.80 ± 0.41	19.65 ± 0.70	19.74 ± 0.46
TIP4P-D	Na^+	18.06 ± 0.94	18.17 ± 0.92	18.14 ± 0.95	18.12 ± 0.82	18.33 ± 0.94
	K^+	17.89 ± 0.46	18.03 ± 0.49	17.85 ± 0.47	17.96 ± 0.49	17.78 ± 0.46
	NaCl	18.60 ± 0.82	18.56 ± 0.70	18.64 ± 0.82	18.64 ± 0.74	18.47 ± 0.77
	KCl	18.24 ± 0.51	18.11 ± 0.40	18.15 ± 0.46	18.22 ± 0.53	18.13 ± 0.46
SPC/E	Na^+	19.22 ± 0.98	19.41 ± 0.82	18.30 ± 1.11	18.95 ± 1.44	19.36 ± 0.76
	K^+	20.12 ± 0.51	20.12 ± 0.51	20.18 ± 0.49	20.14 ± 0.51	20.15 ± 0.48
	NaCl	19.86 ± 0.92	19.75 ± 0.88	19.89 ± 0.79	19.90 ± 0.91	19.94 ± 0.99
	KCl	20.53 ± 0.46	20.53 ± 0.48	20.49 ± 0.54	20.44 ± 0.51	20.43 ± 0.43
OPC	Na^+	17.47 ± 0.88	17.15 ± 1.05	17.49 ± 0.96	17.51 ± 0.90	17.55 ± 0.86
	K^+	17.65 ± 0.54	17.79 ± 0.46	17.78 ± 0.52	17.70 ± 0.60	17.74 ± 0.53
	NaCl	17.91 ± 0.78	17.74 ± 1.00	17.89 ± 0.77	17.80 ± 0.90	17.86 ± 0.72
	KCl	18.12 ± 0.50	18.15 ± 0.52	18.17 ± 0.44	18.26 ± 0.54	18.16 ± 0.55

Table S8: Mean values of roll (and standard deviations) of 1RNA duplex calculated over complete multiple simulations using different setup of counterions. The first two base pairs from each end terminal of the strands were excluded to prevent end effects. The roll of crystal structure is 9.96°.

Water model	Ions	Roll [°]				
		Sim #1	Sim #2	Sim #3	Sim #4	Sim #5
TIP3P	Na^+	10.76 ± 0.49	10.67 ± 0.49	10.59 ± 0.50	10.59 ± 0.65	10.61 ± 0.43
	K^+	11.07 ± 0.33	11.10 ± 0.28	11.05 ± 0.28	11.11 ± 0.31	11.06 ± 0.28
	NaCl	11.30 ± 0.68	11.49 ± 1.17	11.25 ± 0.50	11.14 ± 0.58	11.12 ± 0.53
	KCl	11.56 ± 0.30	11.46 ± 0.27	11.48 ± 0.25	11.44 ± 0.44	11.44 ± 0.28
TIP4PD	Na^+	10.37 ± 0.54	10.41 ± 0.53	10.41 ± 0.56	10.40 ± 0.48	10.51 ± 0.56
	K^+	10.20 ± 0.28	10.27 ± 0.29	10.17 ± 0.29	10.22 ± 0.14	10.13 ± 0.28
	NaCl	10.89 ± 0.44	10.66 ± 0.41	10.73 ± 0.47	10.71 ± 0.44	10.63 ± 0.46
	KCl	10.43 ± 0.29	10.34 ± 0.25	10.37 ± 0.29	10.40 ± 0.32	10.36 ± 0.28
SPC/E	Na^+	11.21 ± 0.58	11.27 ± 0.49	10.85 ± 0.60	11.09 ± 0.72	11.26 ± 0.44
	K^+	11.69 ± 0.31	11.70 ± 0.52	11.73 ± 0.30	11.71 ± 0.31	11.71 ± 0.29
	NaCl	11.58 ± 0.54	11.52 ± 0.88	11.59 ± 0.47	11.60 ± 0.53	11.64 ± 0.59
	KCl	11.96 ± 0.27	11.96 ± 0.29	11.94 ± 0.33	11.91 ± 0.31	11.90 ± 0.26
OPC	Na^+	10.06 ± 0.51	9.90 ± 0.59	10.05 ± 0.57	10.06 ± 0.54	10.08 ± 0.51
	K^+	10.13 ± 0.33	10.22 ± 0.28	10.21 ± 0.31	10.16 ± 0.36	10.19 ± 0.32
	NaCl	10.34 ± 0.44	10.24 ± 0.58	10.33 ± 0.45	10.27 ± 0.53	10.31 ± 0.43
	KCl	10.44 ± 0.30	10.47 ± 0.31	10.45 ± 0.30	10.50 ± 0.32	10.46 ± 0.33

Table S9: Mean values of twist (and standard deviations) of 1RNA duplex calculated over complete multiple simulations using different setup of counterions. The first two base pairs from each end terminal of the strands were excluded to prevent end effects. The twist of crystal structure is 30.55°.

Water model	Ions	Twist [°]				
		Sim #1	Sim #2	Sim #3	Sim #4	Sim #5
TIP3P	Na^+	29.98 ± 0.12	30.00 ± 0.12	30.00 ± 0.10	30.05 ± 0.17	30.01 ± 0.10
	K^+	30.12 ± 0.13	30.11 ± 0.08	30.10 ± 0.09	30.11 ± 0.10	30.12 ± 0.09
	NaCl	30.06 ± 0.15	29.96 ± 1.17	30.03 ± 0.19	30.06 ± 0.12	30.07 ± 0.11
	KCl	30.14 ± 0.19	30.17 ± 0.10	30.17 ± 0.10	30.44 ± 1.00	30.16 ± 0.07
TIP4P-D	Na^+	30.00 ± 0.22	30.00 ± 0.26	30.00 ± 0.17	30.00 ± 0.22	29.97 ± 0.20
	K^+	29.89 ± 0.15	29.84 ± 0.14	29.87 ± 0.14	29.87 ± 0.14	29.89 ± 0.13
	NaCl	30.00 ± 0.26	30.02 ± 0.19	30.05 ± 0.22	30.01 ± 0.16	30.08 ± 0.19
	KCl	29.96 ± 0.19	29.93 ± 0.13	29.93 ± 0.14	29.91 ± 0.14	29.93 ± 0.14
SPC/E	Na^+	30.44 ± 0.83	30.21 ± 0.16	32.47 ± 1.45	31.49 ± 1.62	30.22 ± 0.13
	K^+	30.28 ± 0.13	30.28 ± 0.12	30.29 ± 0.11	30.29 ± 0.12	30.29 ± 0.12
	NaCl	30.26 ± 0.17	30.25 ± 0.17	30.26 ± 0.79	30.26 ± 0.15	30.27 ± 0.15
	KCl	30.29 ± 0.12	30.30 ± 0.13	30.33 ± 0.16	30.33 ± 0.14	30.33 ± 0.13
OPC	Na^+	30.17 ± 0.20	30.62 ± 1.13	30.11 ± 0.18	30.13 ± 0.17	30.12 ± 0.16
	K^+	30.15 ± 0.13	30.16 ± 0.12	30.16 ± 0.14	30.16 ± 0.13	30.17 ± 0.13
	NaCl	30.22 ± 0.18	30.23 ± 0.18	30.24 ± 0.20	30.20 ± 0.19	30.24 ± 0.18
	KCl	30.25 ± 0.14	30.28 ± 0.17	30.24 ± 0.12	30.21 ± 0.12	30.23 ± 0.12

Table S10: Mean values of propeller (and standard deviations) of 1RNA duplex calculated over complete multiple simulations using different setup of counterions. The first two base pairs from each end terminal of the strands were excluded to prevent end effects. The propeller of crystal structure is -18.82°.

Water model	Ions	Propeller [°]				
		Sim #1	Sim #2	Sim #3	Sim #4	Sim #5
TIP3P	Na^+	-16.90 ± 0.49	-16.98 ± 0.26	-16.97 ± 0.29	-16.66 ± 0.78	-17.03 ± 0.30
	K^+	-17.04 ± 0.23	-17.11 ± 0.18	-17.12 ± 0.18	-17.03 ± 0.31	-17.06 ± 0.23
	NaCl	-17.36 ± 0.30	-17.20 ± 0.41	-17.30 ± 0.38	-17.30 ± 0.34	-17.33 ± 0.34
	KCl	-17.02 ± 0.62	-17.27 ± 0.20	-17.30 ± 0.18	-16.69 ± 0.80	-17.22 ± 0.17
TIP4P-D	Na^+	-17.09 ± 0.51	-17.05 ± 0.48	-17.07 ± 0.52	-17.04 ± 0.65	-17.15 ± 0.53
	K^+	-16.83 ± 0.29	-16.49 ± 0.35	-16.82 ± 0.30	-16.77 ± 0.41	-16.84 ± 0.26
	NaCl	-17.23 ± 0.57	-17.17 ± 0.58	-17.37 ± 0.52	-17.32 ± 0.46	-17.38 ± 0.51
	KCl	-17.04 ± 0.26	-16.92 ± 0.27	-16.97 ± 0.28	-16.85 ± 0.29	-16.88 ± 0.34
SPC/E	Na^+	-16.87 ± 0.79	-17.46 ± 0.42	-16.53 ± 0.94	-16.75 ± 0.75	-17.52 ± 0.41
	K^+	-17.43 ± 0.19	-17.47 ± 0.18	-17.49 ± 0.15	-17.47 ± 0.17	-17.49 ± 0.17
	NaCl	-17.71 ± 0.40	-17.81 ± 0.44	-17.63 ± 0.48	-17.73 ± 0.44	-17.76 ± 0.45
	KCl	-17.46 ± 0.29	-17.48 ± 0.30	-17.50 ± 0.25	-17.60 ± 0.19	-17.54 ± 0.26
OPC	Na^+	-17.48 ± 0.57	-16.95 ± 0.83	-17.22 ± 0.48	-17.23 ± 0.39	-17.03 ± 0.49
	K^+	-17.25 ± 0.27	-17.27 ± 0.24	-17.29 ± 0.25	-17.06 ± 0.48	-17.28 ± 0.25
	NaCl	-17.69 ± 0.54	-17.59 ± 0.49	-17.60 ± 0.49	-17.57 ± 0.54	-17.54 ± 0.48
	KCl	-17.44 ± 0.30	-17.40 ± 0.29	-17.48 ± 0.23	-17.34 ± 0.31	-17.49 ± 0.21

Table S11: Mean values of helical parameters (and standard deviations) of CG duplex calculated over complete multiple simulations using different setup of counterions. The first two base pairs from each end terminal of the strands were excluded to prevent end effects.

Water model	Ions	Helical parameter [°]			
		Inclination	Roll	Twist	Propeller
TIP3P	Na^+	11.77 ± 1.00	6.61 ± 0.61	30.21 ± 0.21	-10.81 ± 0.93
	K^+	12.94 ± 0.64	7.33 ± 0.38	30.58 ± 0.97	-12.00 ± 0.56
	NaCl	12.55 ± 0.84	7.10 ± 0.55	30.47 ± 0.30	-11.55 ± 0.70
	KCl	13.55 ± 0.50	7.72 ± 0.32	30.78 ± 0.14	-12.47 ± 0.43
TIP4P-D	Na^+	12.49 ± 0.8	7.01 ± 0.51	30.25 ± 0.23	-10.94 ± 0.79
	K^+	12.43 ± 0.68	6.97 ± 0.42	30.30 ± 0.22	-11.16 ± 0.61
	NaCl	12.90 ± 0.91	7.28 ± 0.56	30.36 ± 0.22	-11.28 ± 0.73
	KCl	12.77 ± 0.56	7.19 ± 0.34	30.96 ± 0.19	-11.42 ± 0.52
SPC/E	Na^+	13.94 ± 0.90	7.95 ± 0.57	30.73 ± 0.23	-12.59 ± 0.84
	K^+	14.90 ± 0.69	8.57 ± 0.44	31.13 ± 0.20	-13.50 ± 0.54
	NaCl	14.44 ± 0.86	8.28 ± 0.55	30.84 ± 0.22	-13.01 ± 0.77
	KCl	15.17 ± 0.59	8.76 ± 0.38	31.20 ± 0.16	-13.73 ± 0.49
OPC	Na^+	12.01 ± 0.86	16.75 ± 0.53	30.32 ± 0.23	-10.97 ± 0.68
	K^+	12.39 ± 0.62	6.98 ± 0.38	30.49 ± 0.19	-11.52 ± 0.55
	NaCl	12.42 ± 0.80	7.01 ± 0.50	30.42 ± 0.21	-11.23 ± 0.66
	KCl	12.94 ± 0.56	7.34 ± 0.34	30.65 ± 0.14	-11.97 ± 0.43

Table S12: Mean values of helical parameters (and standard deviations) and base pair fraying of studied 1RNA calculated over complete multiple simulations using different force fields. For calculation of helical parameters, the first two base pairs from each end terminal of the strands were excluded to prevent end effects. The helical parameters of crystal structure are as follows: inclination is 18.75 °, roll is 9.96°, twist is 30.55° and propeller is -18.82°. The stability of terminal base pairs was calculated for base pairs 1-28 and 14-15, respectively.

Force field	Inclination [°]		
	Sim #1	Sim #2	Sim #3
DESRES	18.26 ± 0.58	18.20 ± 0.51	18.19 ± 0.53
Chen-Garcia	12.88 ± 0.80	13.02 ± 1.15	12.91 ± 0.98
ROC	15.22 ± 1.36	15.63 ± 1.09	15.94 ± 1.26
OL3	18.12 ± 0.50	18.15 ± 0.52	18.16 ± 0.54
	Roll [°]		
DESRES	10.08 ± 0.36	10.08 ± 0.32	10.05 ± 0.35
Chen-Garcia	7.20 ± 0.50	7.27 ± 0.68	7.28 ± 0.60
ROC	8.82 ± 0.79	9.02 ± 0.64	9.01 ± 0.68
OL3	10.44 ± 0.30	10.47 ± 0.31	10.46 ± 0.33
	Twist [°]		
DESRES	29.26 ± 0.27	29.66 ± 0.20	29.62 ± 0.20
Chen-Garcia	29.42 ± 0.43	29.35 ± 0.44	29.94 ± 0.56
ROC	31.00 ± 1.17	30.77 ± 0.58	30.51 ± 1.40
OL3	30.25 ± 0.14	30.28 ± 0.17	30.23 ± 0.12
	Propeller [°]		
DESRES	-16.19 ± 0.44	-16.35 ± 0.25	-16.16 ± 0.45
Chen-Garcia	-11.81 ± 0.63	-11.65 ± 0.63	-11.36 ± 0.74
ROC	-14.42 ± 1.07	-14.28 ± 0.75	-14.28 ± 0.60
OL3	-17.44 ± 0.30	-17.40 ± 0.29	-17.49 ± 0.21
	Stability of terminal base pairs [%]		
	Base pair	Base pair	Base pair

	1-28	14-15	1-28	14-15	1-28	14-15
DESRES	63.68	68.82	76.43	77.30	60.17	76.40
Chen-Garcia	92.76	90.52	53.32	83.22	87.55	27.88
ROC	8.50	4.64	11.78	1.85	16.09	6.38
OL3	88.66	82.83	73.12	87.85	87.61	88.96

Table S13: Mean values of helical parameters (and standard deviations) and base pair fraying of studied 1QC0 calculated over complete multiple simulations using different force fields. For calculation of helical parameters, the first two base pairs from each end terminal of the strands were excluded to prevent end effects. The helical parameters of crystal structure are as follows: inclination is 15.15°, roll is 8.14°, twist is 32.26° and propeller is -12.52°. The stability of terminal base pairs was calculated for base pairs 1-20 and 10-11, respectively.

Force field	Inclination [°]		
	Sim #1	Sim #2	Sim #3
DESRES	14.70 ± 0.70	14.79 ± 0.72	14.74 ± 0.76
Chen-Garcia	9.63 ± 1.01	9.88 ± 1.21	9.90 ± 1.10
ROC	11.31 ± 2.17	11.43 ± 2.22	11.18 ± 2.08
OL3	14.04 ± 0.69	14.11 ± 0.64	14.36 ± 0.77
	Roll [°]		
DESRES	7.74 ± 0.44	7.80 ± 0.43	7.75 ± 0.47
Chen-Garcia	5.10 ± 0.47	5.19 ± 0.58	5.23 ± 0.50
ROC	6.05 ± 1.30	6.18 ± 1.29	5.96 ± 1.22
OL3	7.56 ± 0.43	7.60 ± 0.39	7.74 ± 0.44
	Twist [°]		
DESRES	28.87 ± 0.36	28.87 ± 0.44	28.81 ± 0.36
Chen-Garcia	28.09 ± 0.58	28.07 ± 0.48	28.55 ± 1.54
ROC	29.06 ± 0.98	29.10 ± 0.88	28.73 ± 2.48
OL3	29.35 ± 0.40	29.41 ± 0.22	29.41 ± 0.40
	Propeller [°]		
DESRES	-11.56 ± 0.54	-11.61 ± 0.53	-11.56 ± 0.52
Chen-Garcia	-5.52 ± 0.48	-5.56 ± 0.60	-5.54 ± 0.49
ROC	-9.93 ± 1.61	-10.08 ± 1.52	-9.81 ± 1.66
OL3	-11.95 ± 0.70	-12.00 ± 0.60	-12.03 ± 0.79
	Stability of terminal base pairs [%]		
	Base pair	Base pair	Base pair

	1-20	10-11	1-20	10-11	1-20	10-11
DESRES	95.82	94.25	95.99	93.83	96.17	93.79
Chen-Garcia	99.50	99.19	99.49	99.16	99.51	99.06
ROC	95.98	96.63	95.97	92.68	95.51	93.80
OL3	96.27	94.94	96.63	95.09	96.41	94.41

Table S14: Mean values of helical parameters (and standard deviations) and base pair fraying of studied 2GBH calculated over complete multiple simulations using different force fields. For calculation of helical parameters, the first two base pairs from each end terminal of the strands were excluded to prevent end effects. The helical parameters of NMR structure were calculated as an average value over all frames and are as follows: inclination is 12.12°, roll is 6.21°, twist is 30.20° and propeller is -10.25°. The stability of terminal base pairs was calculated for base pairs 1-16 and 8-9, respectively.

Force field	Inclination [°]		
	Sim #1	Sim #2	Sim #3
DESRES	14.96 ± 0.76	15.12 ± 0.68	15.03 ± 0.70
Chen-Garcia	13.72 ± 1.26	13.69 ± 1.48	13.69 ± 1.46
ROC	12.82 ± 1.86	12.87 ± 1.51	12.79 ± 1.62
OL3	15.79 ± 0.96	15.34 ± 1.30	15.61 ± 0.57
	Roll [°]		
DESRES	8.02 ± 0.49	8.12 ± 0.41	8.09 ± 0.34
Chen-Garcia	7.53 ± 0.85	7.54 ± 0.92	7.51 ± 0.91
ROC	7.18 ± 1.14	7.29 ± 0.92	7.19 ± 1.02
OL3	8.66 ± 0.52	8.43 ± 0.86	8.57 ± 0.34
	Twist [°]		
DESRES	31.51 ± 0.41	31.52 ± 0.30	31.50 ± 0.44
Chen-Garcia	32.08 ± 0.65	32.04 ± 0.67	31.87 ± 0.80
ROC	32.63 ± 0.78	32.95 ± 0.92	32.79 ± 1.06
OL3	31.90 ± 0.37	32.07 ± 1.39	31.96 ± 0.26
	Propeller [°]		
DESRES	-10.84 ± 0.80	-10.95 ± 0.37	-10.94 ± 0.27
Chen-Garcia	-9.22 ± 0.70	-9.29 ± 0.65	-9.22 ± 0.68
ROC	-10.63 ± 0.84	-11.11 ± 0.98	-10.86 ± 0.85
OL3	-11.25 ± 0.58	-11.15 ± 0.50	-11.36 ± 0.33
	Stability of terminal base pairs [%]		

	Base pair		Base pair		Base pair	
	1-16	8-9	1-16	8-9	1-16	8-9
DESRES	96.49	62.64	96.44	64.22	96.88	59.65
Chen-Garcia	99.50	35.50	99.52	46.08	99.49	60.14
ROC	95.92	2.06	96.72	0.71	96.89	0.71
OL3	97.69	52.77	97.92	43.73	97.81	58.40

Table S15: χ^2_{NOE}values of nuclear Overhauser effect intensities calculated over complete multiple simulations of 2GBH duplex using different force fields.

Force field	χ^2		
	Sim #1	Sim #2	Sim #3
DESRES	1.58	1.58	1.59
Chen-Garcia	1.52	1.52	1.53
ROC	1.64	1.66	1.66
OL3	1.58	1.59	1.59

Supporting Figures

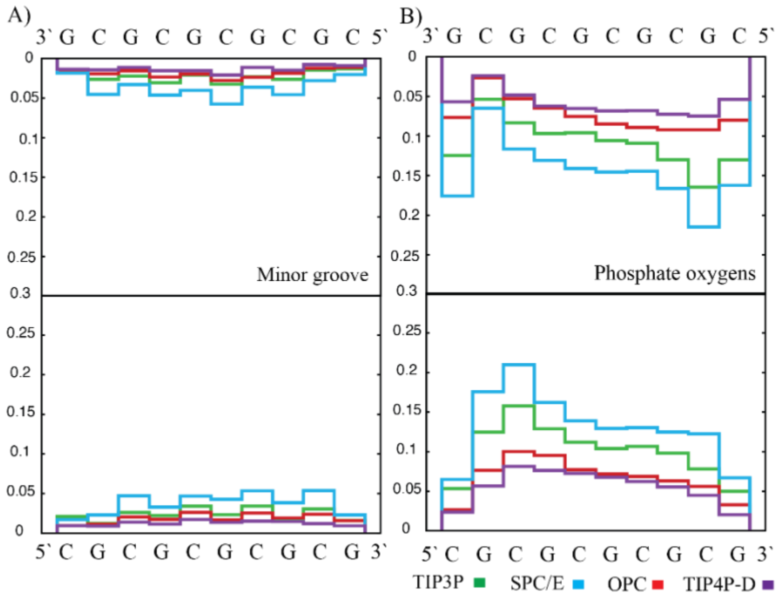

Figure S1. Ion occupancy of each residue in the sequence of the CG duplex in net-neutral simulations using small box. The lower part corresponds to residues from the 5'- to 3'- ends (from left to right), while the top part describes residues from the other strand in reverse order. Average number of ions A) into minor groove, and B) close to phosphate oxygens. The occupancies are calculated within distance of 3.5 Å, which correspond to ions located within first solvation sphere.

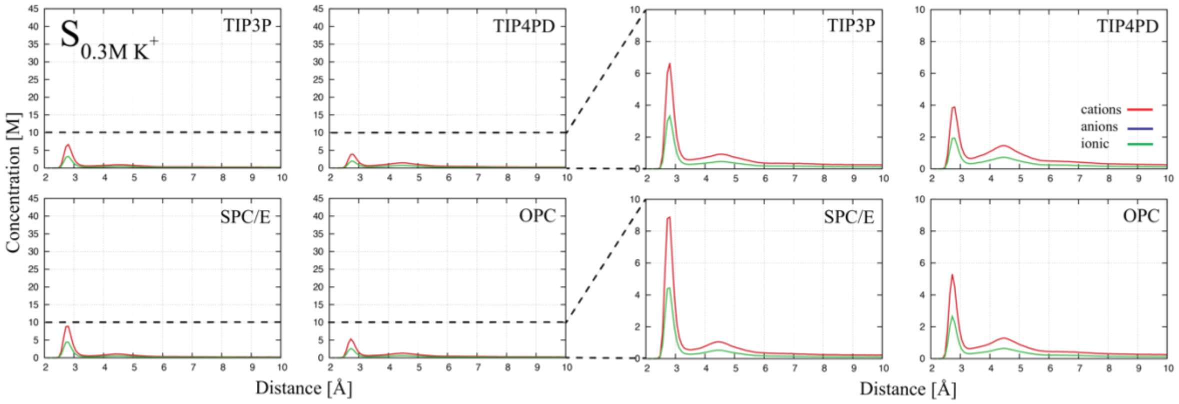

Figure S2: Radial concentration profiles of ions calculated for net-neutral S-box simulations of CG duplex.

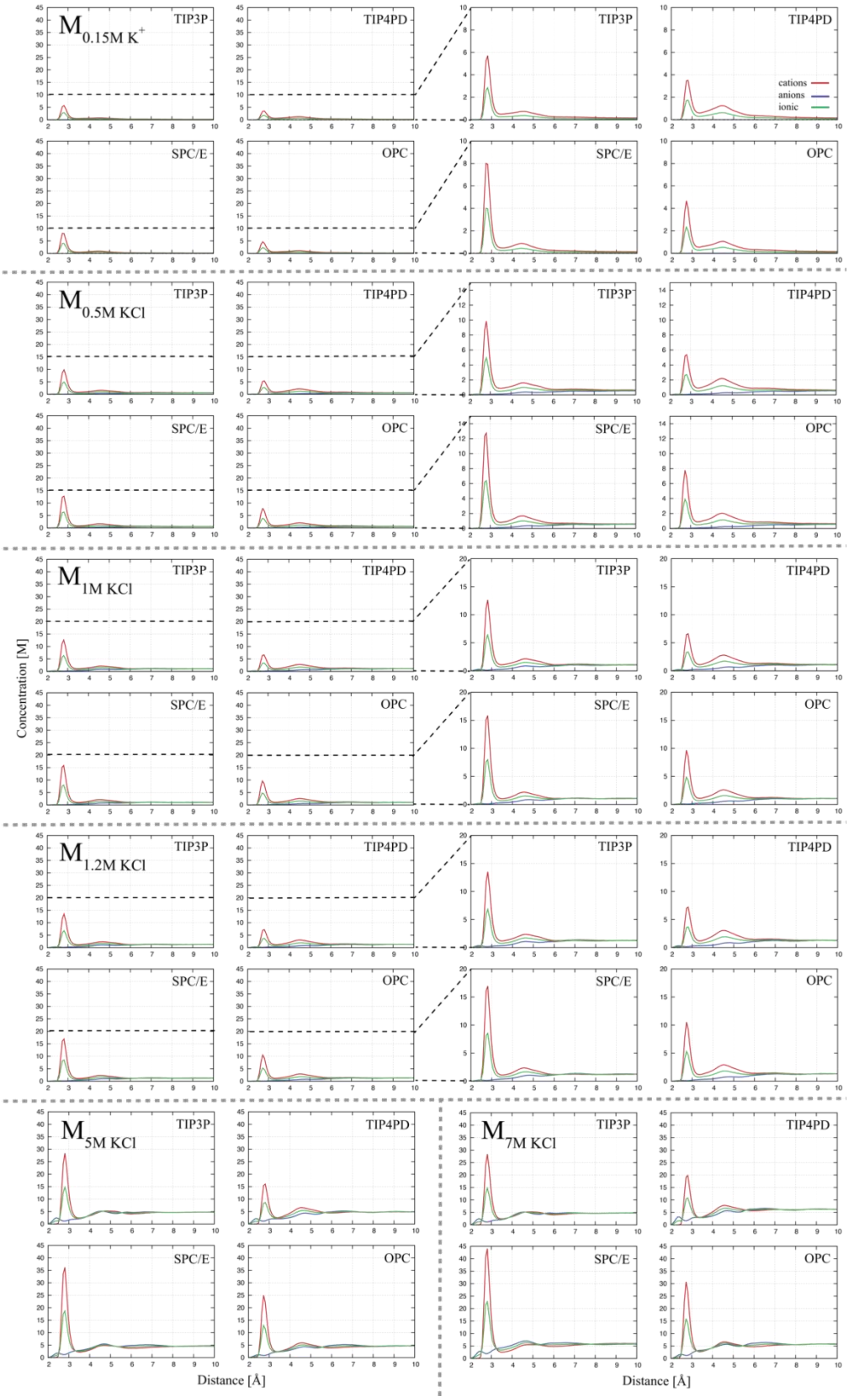

Figure S3: Radial concentration profiles of ions calculated for M-box simulations of CG duplex.

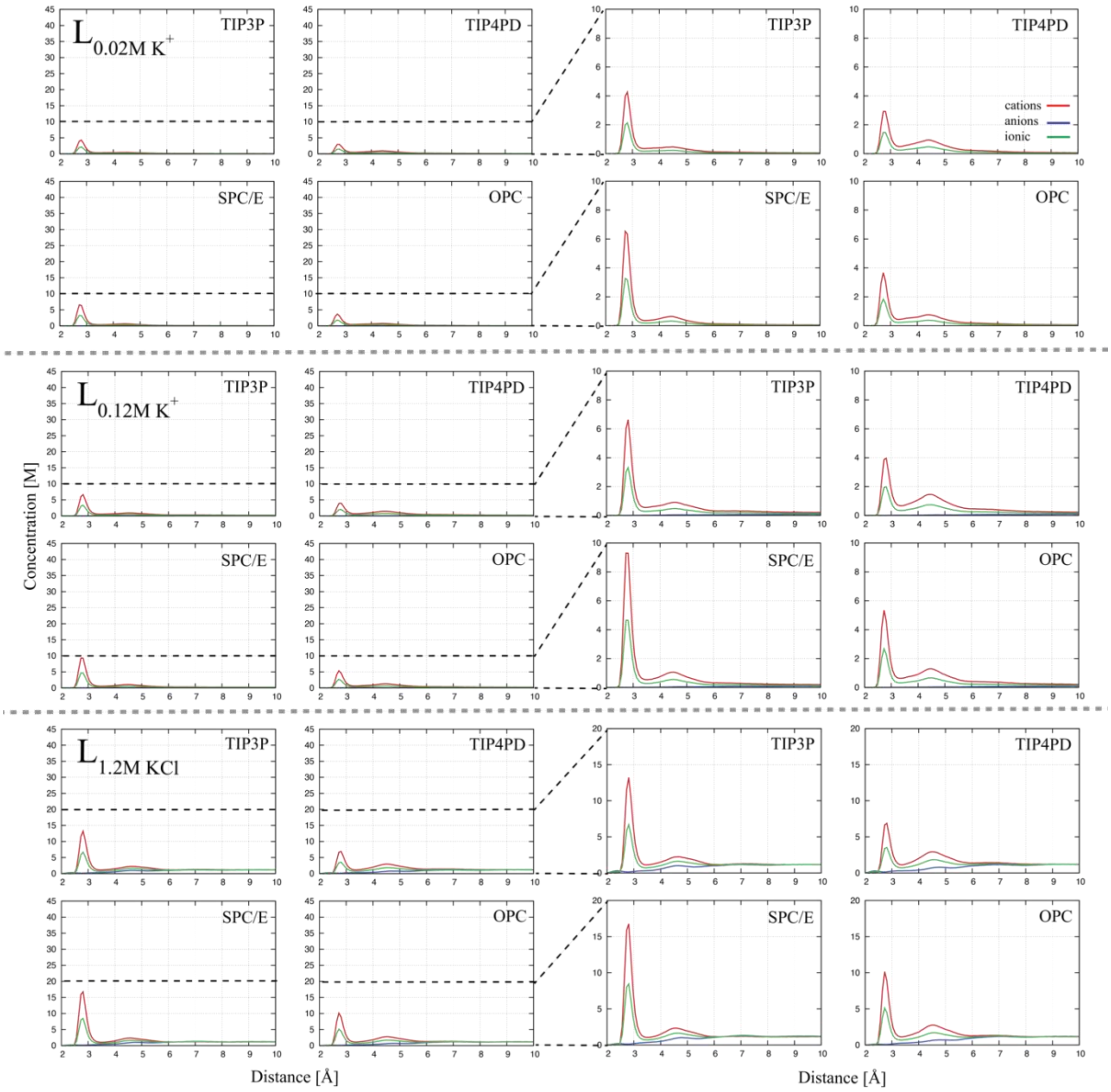

Figure S4: Radial concentration profiles of ions calculated for L-box simulations of CG duplex.

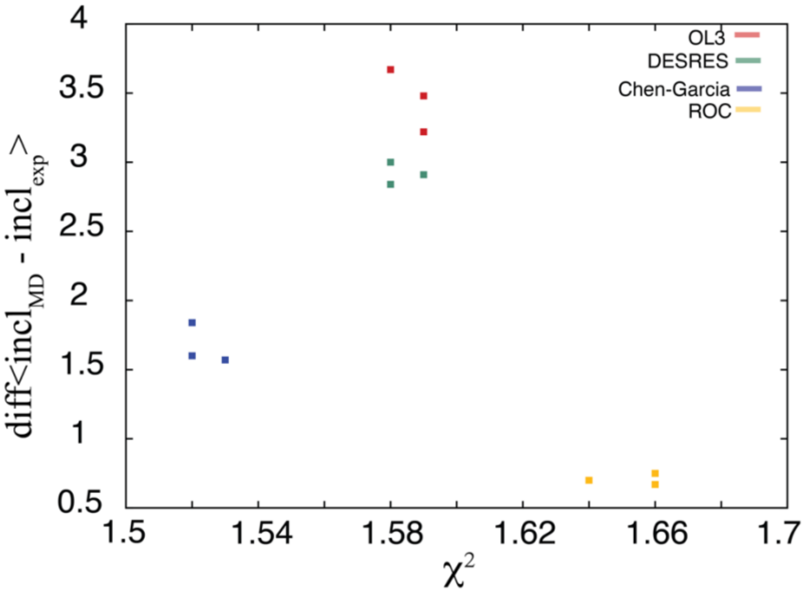

Figure S5: The correlation between the total χ^2_{NOE} values and the diference between the inclination values obtained from the 2GBH simulations (using different force fields) and the experimental inclination value. The experimental inclination value was calculated as the average value over all NMR frames.
